# Fibrillin-1 Orchestrates a Pro-senescent Niche Driving Peritubular Endothelial Senescence via ZEB1/endothelin-1/β-catenin Signaling

**DOI:** 10.7150/ijbs.133521

**Published:** 2026-05-29

**Authors:** Junxin Huang, Xiaoyao Zhang, Zifu Yao, Yuxi Zhang, Di Huang, Yongsi Liu, Fan Fan Hou, Youhua Liu, Li Li

**Affiliations:** 1 State Key Laboratory of Multi-organ Injury Prevention and Treatment, National Clinical Research Center for Kidney and Urological Diseases, Division of Nephrology, Nanfang Hospital, Southern Medical University, Guangzhou, China.; 2 Guangdong Provincial Key Laboratory of Renal Failure Research, Guangdong Provincial Institute of Nephrology, Guangzhou, China.; 3 Light Innovation Technology Ltd., Shenzhen, China.

**Keywords:** fibrillin-1, pro-senescent niche, endothelial senescence, microvascular rarefaction, chronic kidney disease

## Abstract

Microvascular rarefaction is a predominant pathological hallmark of chronic kidney disease (CKD), functioning simultaneously as a catalyst and consequence of progressive renal compromise. Although endothelial senescence constitutes a cardinal mediator of microvascular attrition in CKD, its upstream regulatory mechanism remains elusive. Here, using integrated single-cell/spatial transcriptomics, decellularized scaffold modeling, diverse murine CKD models, vascular ultrasonography, and tissue-clearing-enabled 3D imaging, we identify fibrillin-1 (FBN1), a core constituent of the fibrogenic niche, as an architect of a pro-senescent microenvironment that directly triggers endothelial senescence. Mechanistically, FBN1 upregulates the transcription factor ZEB1, which binds to the *EDN1* promoter to enhance endothelin-1 (ET-1) transcription, thereby activating the ET-1/β-catenin signaling axis to execute cellular senescence. This cascade is abolished by *ZEB1* knockdown, ET-1 receptor antagonism, or β-catenin inhibition. Importantly, tubule-specific *Fbn1* deletion suppresses endothelial senescence, attenuates capillary rarefaction, and ameliorates renal function across CKD models. Our study unveils the FBN1/ZEB1/ET-1/β-catenin axis as a spatially organized signaling pathway linking to endothelial senescence, demonstrating how matrix-embedded components actively perpetuate pathogenesis by orchestrating stable pathological microenvironments. These findings provide a conceptual framework for CKD-associated vascular deterioration and highlight microenvironmental reprogramming as a therapeutic paradigm.

## Introduction

Chronic kidney disease (CKD) affects approximately 10% of the global population and is projected to become the world's fifth leading cause of death by 2040 1-3. The absence of effective therapies sustains significant economic, healthcare, and societal burdens 4-6. In 2025, the World Health Organization designated kidney disease as a major non-communicable disease of global priority 7, heightening the urgency to elucidate CKD pathogenesis and identify therapeutic targets.

Renal fibrosis constitutes the final common pathway for progression to end-stage renal disease across diverse CKD etiologies, pathologically characterized by disrupted extracellular matrix (ECM) equilibrium with excessive deposition outweighing degradation capacity 8, 9. This maladaptive remodeling converges upon four core histopathological hallmarks: microvascular rarefaction, tubular epithelial atrophy, fibroblast activation, and inflammatory cell infiltration 10, 11. Mechanistically, microvascular rarefaction is intimately linked to endothelial senescence. Senescent endothelial cells exhibit multi-faceted dysfunction characterized by compromised vascular integrity, disrupted endothelial barrier properties, and diminished microcirculatory perfusion. These alterations collectively establish a self-reinforcing ischemic microenvironment that both amplifies pre-existing rarefaction and accelerates CKD progression 12-16. Critically, the upstream molecular effectors orchestrating endothelial senescence within the CKD milieu remain poorly defined.

Profound remodeling of the tissue microenvironment governs the initiation and progression of renal fibrosis. Mounting evidence indicates that ECM components function not as inert structural scaffolds but as dynamic signaling organizers, constructing specialized fibrogenic niches to actively direct cellular fate decisions 11, 17-19. Specifically, ECM proteins generate a pro-fibrotic, pro-inflammatory, oxidatively stressed, and anti-angiogenic environment that activates multiple signaling pathways, particularly integrin-dependent and Toll-like receptor-mediated cascades, to precisely coordinate cellular behavior and accelerate CKD progression 20-24. Notably, fibrillin-1 (FBN1), a high-molecular-weight, multidomain ECM glycoprotein, serves as a central architect within fibrogenic niche. Its distinctive structural configuration enables multifunctional signal integration, playing essential roles in microenvironment organization and cellular programming 24-26. Our prior work demonstrates FBN1 upregulation across diverse CKD models, where it engineers pro-apoptotic fibrogenic microenvironments, directs endothelial fate decisions, and drives capillary rarefaction 24. Nevertheless, whether FBN1 directly regulates endothelial senescence and through what molecular mechanisms remains unknown.

In this study, we establish the ECM protein FBN1 as a central orchestrator of endothelial senescence through pathogenic niche formation. Our findings comprehensively delineate how FBN1 constructs a pro-senescent microenvironment, activating the ZEB1/endothelin-1/β-catenin signaling axis to drive endothelial senescence and capillary rarefaction, thereby advancing CKD progression. This work reveals a novel pathogenic mechanism wherein matrix-mediated senescence induction directly propagates renal microvascular attrition. Consequently, targeted disruption of FBN1 or its pathogenic signaling cascade offers a therapeutically actionable strategy to ameliorate microvascular dysfunction and mitigate fibrotic progression in CKD.

## Materials and Methods

### Single cell RNA sequencing and data processing

Transcriptomic data integrated in this study were derived from two sources. First, we generated single-cell RNA sequencing (scRNA-seq) data from a unilateral ischemia-reperfusion injury (UIRI) mouse model and two corresponding sham control groups in our laboratory. Second, publicly available scRNA-seq datasets previously published were obtained from public repositories 27, 28, as described in Supplementary Table 1. To ensure analytical consistency, all datasets were uniformly processed from raw FASTQ files using a standardized pipeline. Raw sequencing data were processed with Cell Ranger (v8.1.0, 10x Genomics). A reference genome was built based on the *Mus musculus* GRCm39.105 annotation, and sequence alignment was performed strictly following the official 10x Genomics guidelines (https://www.10xgenomics.com/support/software/cell-ranger/downloads/cr-ref-build-steps). Unique molecular identifier (UMI) counting and cell barcode assignment were subsequently conducted, yielding gene expression matrices for downstream analyses.

Following initial preprocessing, scRNA-seq data were analyzed using Seurat (v5.3.0) 29. Stringent quality control (QC) was performed to exclude low-quality cells: cells with the number of expressed genes < 800 or > 6,000, mitochondrial gene proportion > 30%, ribosomal gene proportion > 10%, or gene complexity score (log10GenesPerUMI) outside the range of 0.8-0.95 were removed. Potential doublets were identified and eliminated using scDblFinder (v1.22.0) 30, and only high-confidence single cells were retained for subsequent analyses.

After filtering, the data were normalized via the log-normalization method (NormalizeData function) and further scaled using ScaleData to correct for technical variations. The top 2,000 highly variable genes were selected using the FindVariableFeatures function for downstream analysis. Principal component analysis (PCA) was conducted with RunPCA, and batch effects across different samples were corrected using Harmony (v1.2.3) 31, with sample origin as the grouping variable. Based on the Harmony-corrected low-dimensional space, a shared nearest neighbor (SNN) graph was constructed using FindNeighbors, and clustering analysis was performed with the FindClusters function (resolution = 1) to define distinct cell populations.

The following analyses were next carried out. 1) Differential gene analysis. Differential expression analysis between distinct cell populations was performed using the FindAllMarkers function in Seurat 32, with the Wilcoxon rank-sum test (Wilcox test) employed as the statistical method for significance assessment. 2) Senescence scoring analysis. To evaluate the cellular senescence status, we extracted relevant genes from the GO term “Cellular senescence” (GO:0090398) as the signature gene set. The standardized single-cell senescence activity score was calculated using the AddModuleScore function in Seurat, which computes the average expression of senescence-related genes and corrects for background signals to eliminate potential technical biases. 3) Enrichment analysis. To identify significantly altered biological pathways, we performed gene set enrichment analysis (GSEA) on the differentially expressed genes (adjusted *P* < 0.05) 33. Based on the GO Biological Process database of *Mus musculus* (mouse), the false discovery rate (FDR) correction was applied, and pathways with an adjusted *P*-value < 0.05 were considered significantly enriched. 4) Pseudotime analysis. Pseudotime trajectory analysis was conducted using the Slingshot algorithm 34. Based on the Uniform Manifold Approximation and Projection (UMAP) low-dimensional space, the Homeostatic EC subset was designated as the initial state node to reconstruct the endothelial cell differentiation trajectory.

### Spatial transcriptomics sequencing and data processing

The spatial transcriptomic data utilized in this study were integrated and reused from our team's previously published work 21. Quality control, dimensionality reduction, clustering, and region type annotation (spatial resolution: 16 μm) of this dataset had been completed in the original study. Without repeating the aforementioned analytical pipelines, the present study performed further investigations based on the established results of the original analysis.

The deconvolution analysis of spatial transcriptomic data was conducted in accordance with the analytical workflow previously published by our team 21, and the results were consistent with those reported in the prior study. In this research, the newly generated single-cell transcriptomic data were used as the deconvolution reference. While maintaining all original parameter settings unchanged, the endothelial cell subset analysis results identified in the present study were further incorporated to perform more refined deconvolution analysis of endothelial cells.

Considering that senescent cells are mainly distributed in the renal cortical region, cells located in the cortex were first filtered for subsequent correlation analysis. The Pearson correlation coefficient was used to evaluate the expression correlation between different genes at spatial positions in the cortical region. Based on the magnitude of the correlation coefficients, genes significantly correlated with the 9 target genes of key interest in the preliminary study were further screened for in-depth analysis 17. To ensure the statistical reliability of the results, the significance threshold was set at *P* < 0.01.

### Animal models

The renal tubule-specific *Fbn1* knockout mice were generated by crossing *Cdh16*-Cre mice with *Fbn1*-floxed mice, both acquired from Shanghai Model Organisms (Shanghai, China). Additional experimental animals were sourced from Beijing Vital River Laboratory (Beijing, China). All animals were maintained at the Animal Center of Nanfang Hospital, Southern Medical University. Mice were randomly subjected to one of four injury models: unilateral ureteral obstruction (UUO), UIRI, folic acid-induced nephropathy (FA), or aristolochic acid nephropathy (AAN). According to established protocols, the experimental durations were 7 days for UUO 35, 11 days for UIRI 23, 14 days for FA 36, and 28 days for AAN 37. All procedures were conducted in accordance with the NIH Guide for the Care and Use of Laboratory Animals and were approved by the Institutional Animal Care and Use Committee of Nanfang Hospital, Southern Medical University (Protocol #IACUC-LAC-20230704-003).

### Cell culture and treatment

Human umbilical vein endothelial cells (HUVECs) were cultured in high-glucose DMEM medium supplemented with 10% fetal bovine serum. Cells were treated with varying concentrations of recombinant human FBN1 (#10224-FI, R&D Systems) or recombinant human ET-1 (#1160, Tocris) for the indicated durations. In selected experiments, cells were pre-incubated for 1 h with Bosentan (100 nM; HY-A0013, MCE) or ICG-001 (10 μM; HY-14428, MCE), followed by stimulation with FBN1 (50 ng/mL) for 36 h.

### Preparation of the kidney tissue scaffolds

Decellularized kidney tissue scaffolds (KTS) were prepared according to established protocols 20. Porcine kidneys were sectioned into uniformly thick slices (3-4 cm) after euthanasia, whereas mouse kidneys were sliced into consistently thick sections. Tissue slices were subsequently decellularized through sequential treatments with sodium deoxycholate and Triton X-100. HUVECs were then seeded onto the KTS, followed by collection of cell lysates for subsequent analysis.

### Bulk RNA sequencing and data analyses

Bulk RNA sequencing was performed as previously described 38. Total RNA was extracted, quality-controlled, and used for library preparation. Libraries were sequenced on Illumina NovaSeq 6000 and BGI MGISEQ-T7 platforms to generate 150-bp paired-end reads. Following alignment and gene-level quantification, differential expression analysis was conducted using DESeq2. The bioinformatic analyses, including data processing, as well as the generation of heatmaps, volcano plots, and GO/KEGG enrichment plots, were supported by a standardized pipeline from Shanghai Applied Protein Technology, Guangzhou YanCe Gene Co., Ltd., and the online platform (https://www.bioinformatics.com.cn) 39.

### SA-β-gal staining and MitoSOX staining

Cultured cells were stained for β-galactosidase activity using a commercial kit (C0602, Beyotime) according to the manufacturer's protocol. Mitochondrial ROS production was assessed via MitoSOX (M36008, Thermo Fisher) staining according to the manufacturer's instructions.

### Wound-healing assay and tube formation assay

The wound-healing assay was performed to assess cell migration. A uniform scratch was created in a confluent monolayer using a sterile pipette tip. After washing with PBS to remove detached cells and debris, images of the wound area were captured at 0 and 48 h using an inverted microscope. The remaining wound area was quantified with ImageJ software to evaluate migratory capacity. The tube formation assay was performed to assess angiogenic capacity. HUVECs were seeded on Matrigel-coated plates (GL101, Vazyme). The formed tubular networks were imaged using a phase-contrast microscope. Images were quantitatively analyzed with ImageJ software and the Angiogenesis Analyzer plugin.

### Live-cell label-free microscopy

Cells were observed using a live-cell label-free microscopic imaging system (SC3000, ZIRCON). After seeding in culture plates and treatment with FBN1 for 36 h, images were acquired and analyzed for changes in cellular morphology and organelles.

### Western blot analysis

Western blotting was performed according to established protocols 22, 40. Briefly, proteins were extracted and quantified, followed by electrophoretic separation and electroblotting onto PVDF membranes. The membranes were blocked and incubated with primary antibodies and corresponding HRP-conjugated secondary antibodies (details in Supplementary Table S2). Protein bands were visualized and analyzed with ImageJ software.

### Quantitative real-time PCR

Total RNA was extracted using the TRIzol reagent (#15596018CN, Thermo Fisher). Quantitative PCR was performed on an ABI PRISM 7000 system as previously described 41, with mRNA levels normalized to β-actin. Primer sequences are listed in Supplementary Table S3.

### Isolation and culture of mouse primary renal endothelial cells

Primary renal endothelial cells were isolated via enzymatic digestion and density-gradient centrifugation. Briefly, kidneys were carefully excised, decapsulated, and minced into fragments. The tissue was digested at 37 °C for 30-45 min in a solution containing 1 mg/mL collagenase type IV (#17104019, Gibco) using a tissue dissociator (DSC-410, RWD). The digested suspension was sequentially passed through 70 µm and 40 µm cell strainers, centrifuged at 300 × g, and treated with erythrocyte lysis buffer. Finally, the pellet was resuspended and overlaid onto a pre-formed discontinuous Percoll gradient (25%-50%; #17089101, Cytiva). After centrifugation, the endothelial cell layer collected from the interface was resuspended in complete medium for culture. Endothelial cell identity was validated by immunofluorescence staining for CD31.

### Chromatin immunoprecipitation

Chromatin immunoprecipitation (ChIP) assays were performed using a commercial kit (#9005, Cell Signaling Technology). Briefly, cells were cross-linked with formaldehyde, and chromatin was sheared by sonication. Immunoprecipitation was carried out overnight at 4 °C with antibodies against ZEB1 (Cat No. 21544-1-AP, Proteintech), histone H3 (H3), or normal rabbit IgG, followed by capture with protein A-agarose beads. After washing, elution, reverse cross-linking, and DNA purification, the precipitated DNA was subjected to PCR analysis. Primers for the human *EDN1* promoter were as follows: forward, 5′-ATTCTGGGTGCTCAGTTGTC-3′; reverse, 5′-TGAAGTACACACAGGGCAG-3′.

### siRNA transfection

siRNA-mediated *ZEB1* knockdown was performed using the Rfect Huvec siRNA transfection reagent (#11059, Baidaibio). Briefly, HUVECs were seeded at appropriate density and transfected with siRNA targeting *ZEB1* (sequence: 5′-GCUGUUGUUCUGCCAACAGUU-3′) according to the manufacturer's instructions. Transfection efficiency was confirmed by measuring *ZEB1* mRNA levels via RT-qPCR.

### Immunohistochemical and immunofluorescence staining

Kidney tissues were fixed in 4% formaldehyde for 24 h and embedded in paraffin using standard histological methods. Immunohistochemical and immunofluorescence staining were carried out according to established protocols 21. Antibodies used in this study are listed in Supplementary Table S2.

### Vascular permeability assay

Renal vascular permeability was assessed using fluorescein-conjugated dextran (#74814, Sigma). Mice were injected with FITC-dextran (100 mg/kg) via the tail vein. After 15 min, animals were perfused transcardially with saline, and kidneys were harvested, embedded, and sectioned at 3 μm for immunofluorescence staining of EMCN. Extravasated FITC-dextran was visualized directly by fluorescence microscopy. Signal intensity was quantified to evaluate the degree of microvascular leakage.

### Ultra-resolution ultrasound microscopy of renal microvasculature

Renal microvascular imaging in mice was conducted using an ultra-resolution ultrasound microscopy system (ULTIMUS 9LAB, VINNO). After anesthesia, mice were intravenously administered microbubble contrast agent, immediately followed by 30 s of ultra-resolution microscopy (URM) acquisition. All scans were positioned at the renal transverse plane with optimal vascular visualization. Data were processed using the built-in URM analysis module to generate functional maps of microvascular density, velocity and direction. Quantitative parameters including vessel ratio, complexity level, and perfusion index were subsequently derived.

### Tissue clearing

Tissue samples underwent gradient dehydration in methanol/B1n buffer, followed by sequential washes in dichloromethane and methanol to remove residues, and were subsequently bleached overnight at 4°C in 5% H₂O₂/methanol in the dark. After gradual rehydration to an aqueous phase, immunolabeling was performed by pre-incubation in OmniStain A, followed by 4-day incubations at room temperature with primary antibody and secondary antibody, respectively, with extensive washes in OmniStain B and PBSN buffer between steps. Finally, samples were dehydrated through a methanol gradient, cleared in BABB solution under dark conditions with gentle agitation until optically transparent, and prepared for imaging. Kidney vascular structures were semi-automatically annotated and segmented using the Labkit module in Fiji. The resulting segmentation results were exported into Amira (Thermo Fisher), where global analysis was performed to quantify total vascular volume.

### Statistical analyses

Data are presented as mean ± SEM. Statistical analyses of the data were performed using SPSS 22.0 (SPSS Inc.). Two-group comparisons were analyzed with the unpaired two-tailed t-test (or Mann-Whitney U test for non-normal data). Multi-group comparisons were conducted using one-way ANOVA (or Kruskal-Wallis test), followed by Fisher's LSD test (equal variance) or Dunnett's T3 test (unequal variance) for post-hoc analysis. Differences were considered statistically significant at *P* < 0.05.

## Results

### FBN1 establishes a pro-senescent microenvironment orchestrating endothelial senescence

**Figure 1:**
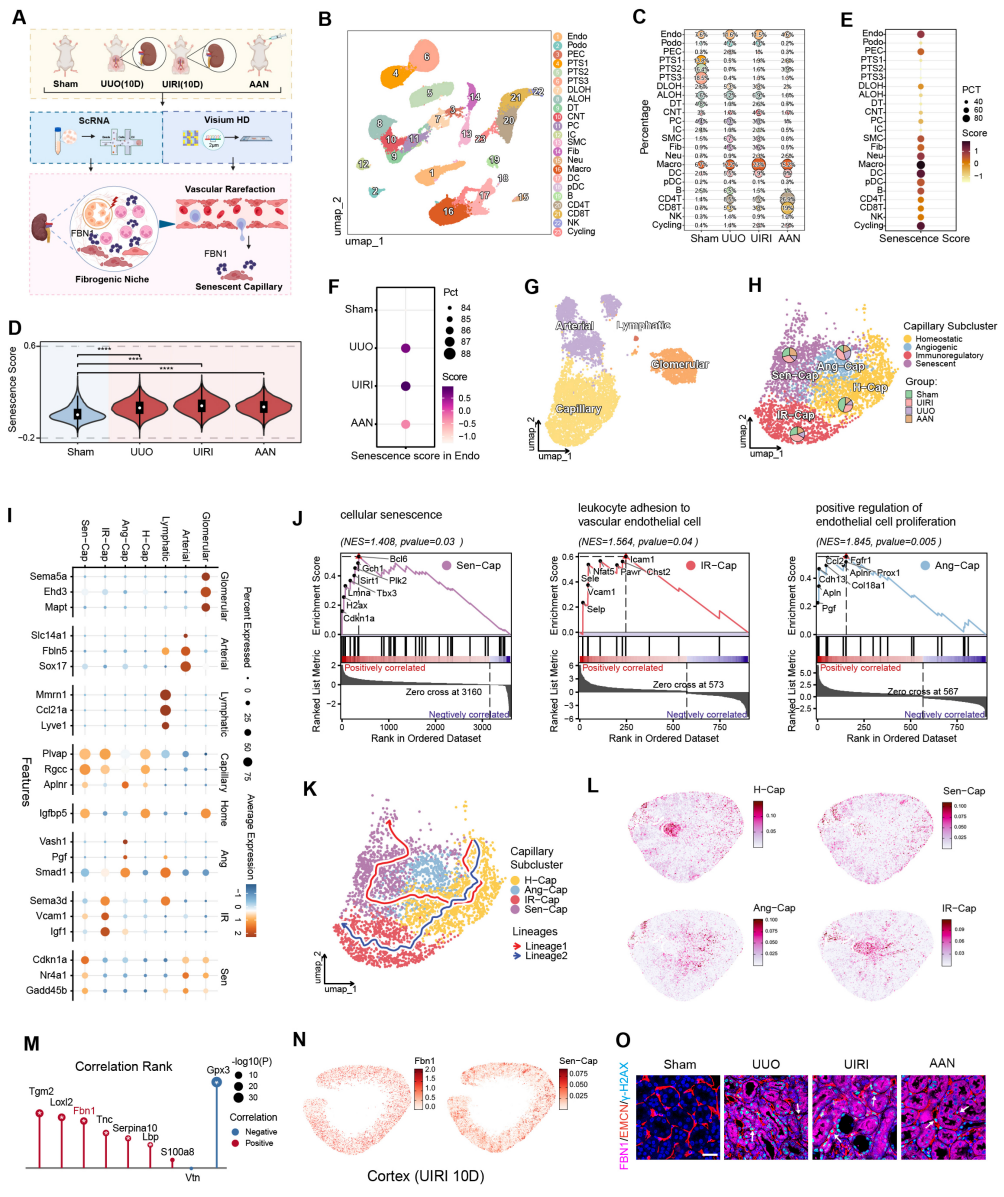
** FBN1 establishes a pro-senescent microenvironment orchestrating endothelial senescence. (A)** Schematic representation of single-cell RNA sequencing (scRNA-seq) and spatial transcriptomics (ST) of kidneys from the Sham, unilateral ureteral obstruction (UUO), unilateral ischemia-reperfusion injury (UIRI) and aristolochic acid nephropathy (AAN) mouse models. **(B)** t-SNE plot illustrating the comprehensive cellular atlas of renal tissue across different chronic kidney disease (CKD) conditions. Major cell populations are annotated and color-coded, including glomerular endothelial cells (GEC), podocytes (Podo), proximal tubules (PT), descending limbs of Henle (DLOH), ascending limbs of Henle (ALOH), distal tubules (DT), connecting tubule cells (CNT), principal cells (PC), intercalated cells (IC), fibroblasts (Fib), smooth muscle cells (SMC), monocytes (Mono), dendritic cells (DC), macrophages (Macro), plasmacytoid dendritic cells (pDC), neutrophils (Neu), B cells (B), CD4⁺ T cells (CD4T), CD8⁺ T cells (CD8T), natural killer cells (NK), and cycling cells (Cycling). Cell identities are directly labeled in the plot. **(C)** Bubble plot illustrating the relative proportions of major renal cell types across Sham, UUO, UIRI and AAN samples. Each dot represents a specific cell type within a given experimental group, with dot size corresponding to its relative abundance. **(D)** Violin plots showing differences in senescence scores across experimental groups. Statistical analysis was performed using the Wilcoxon rank-sum test. ****P < 0.0001 versus Sham. **(E)** Dotplot displaying senescence scores across different renal cell types. Dot color represents the average senescence score, while dot size indicates the proportion of cells contributing to the senescence gene module. **(F)** Dotplot showing senescence scores of endothelial cells across different experimental groups. Dot color indicates the average gene expression level within each region, while dot size represents the proportion of spatial spots expressing the gene. **(G)** UMAP visualization of endothelial cell subpopulations. **(H)** UMAP plot of capillary endothelial cell subtypes. Pie charts depict the relative distribution of each subtype across experimental groups. **(I)** Bubble plot showing the expression patterns of marker genes across endothelial cell subtypes. Dot size represents the proportion of cells expressing the gene, while dot color indicates the scaled average expression level. **(J)** Gene set enrichment analysis (GSEA) showing pathways significantly enriched in each capillary endothelial subtype compared with the homeostatic subtype. **(K)** UMAP-based pseudotime trajectory analysis of capillary endothelial cells inferred using the Slingshot algorithm. Cells are colored by functional subtype, and two major lineage trajectories are overlaid to illustrate state transitions. **(L)** Spatial distribution of endothelial cells in distinct functional states, inferred by RCTD-based deconvolution of Visium HD spatial transcriptomics data. **(M)** Rank plot showing genes are ranked by correlation strength. Positive and negative correlations are indicated in red and blue, respectively. Dot size represents statistical significance (-log10* P*), with *P* < 0.01 marked by an asterisk (*). **(N)** Spatial localization of *Fbn1* and senescent capillary endothelial cells (Sen-Cap) within the renal cortex. **(O)** Representative immunofluorescence images of FBN1, EMCN, and γ-H2AX expression in the renal cortex across different experimental groups. Arrows point to senescent endothelial cells. Scale bar, 25 μm.

The senescence of peritubular endothelial cells represents a common pathological hallmark of CKD, emerging across distinct disease etiologies 16. To investigate the prevalence and dynamics of endothelial senescence during CKD progression, we performed scRNA-seq in three murine models of renal fibrosis, including UUO, UIRI, and AAN 21, 27. This systemic profiling identified core molecular mechanisms linking endothelial cell senescence to microvascular rarefaction. Furthermore, using high-resolution spatial transcriptomics (Visium HD) in the UIRI model, we spatially mapped the localization of senescent endothelial cells within the renal architecture (Figure 1A).

Through integrated cross-model single-cell transcriptomic analysis, we systematically constructed a high-resolution cellular atlas of CKD (Figure 1B-C). Quantitative evaluation of senescence-associated signatures demonstrated a pronounced increase in senescent features across all renal parenchymal cell types in the three disease models. Among parenchymal cells, vascular endothelial cells displayed significantly higher senescence scores compared with other cell types (Figure 1D-F). In-depth subpopulation analysis classified endothelial cells into four subsets with distinct molecular signatures: an arteriolar subset defined by arterial developmental genes (*Fbln5* and *Sox17*) 42, 43, a peritubular subset responsible for solute exchange, a glomerular subset enriched in filtration-related membrane transport genes (*Ehd3*) 44, and a lymphatic subset associated with lymph circulation, marked by lymphatic specific genes (*Mmrn1* and *Ccl21a*) 45, 46 (Figure 1G). Focusing specifically on the peritubular endothelial subset, we further deconvoluted this population into four functionally distinct subtypes: a homeostatic subtype (*Igfbp5*+), an angiogenic subtype (*Vash1*+, *Pgf*+, *Smad*1+) 47, 48, an immunomodulatory subtype (*Sema3d*+, *Vcam1*+, *Igf1*+) 49, 50, and a senescent subtype (*Cdkn1a*+, *Gadd45b*+) 51, 52 (Figure 1H-I). GSEA demonstrated clear functional divergence among these subtypes. Relative to the homeostatic population, the senescent subtype was selectively enriched in cellular senescence pathways, whereas the immunomodulatory subtype showed significant enrichment for leukocyte chemotaxis and adhesion pathways, and the angiogenic subtype specifically activated pathways related to endothelial proliferation (Figure 1J). Building on these functional profiles, trajectory reconstruction by the Slingshot algorithm revealed that homeostatic peritubular endothelial cells could diverge along two distinct differentiation trajectories, transitioning towards either the immunomodulatory or the senescent phenotype (Figure 1K) 34.

To resolve the tissue topography of these divergent subtypes, we performed cell-type deconvolution by integrating our published high-resolution Visium HD spatial transcriptomic dataset 21. The results clearly demonstrated that homeostatic endothelial cells were uniformly distributed throughout the renal tissue, consistent with their functional role in maintaining basal vascular homeostasis. In contrast, angiogenic and immunomodulatory endothelial cells were specifically enriched at the corticomedullary junction. Of note, senescent endothelial cells were predominantly enriched in the cortex, where renal tubules are densely distributed. This spatial colocalization suggests that the local microenvironment of injured tubules may directly contribute to the regulation of endothelial cell senescence (Figure 1L). Given this colocalization, we next sought to define the molecular composition of the senescence-promoting niche by investigating potential interactions between cortical senescent endothelial cells and specific extracellular matrix (ECM) components. We systematically correlated the spatial distribution of senescent endothelial cells in the cortex with the expression of nine key ECM molecules previously identified from our kidney tissue scaffolds (KTS) work 17. The results demonstrated that transglutaminase 2 (*Tgm2*), lysyl oxidase-like 2 (*Loxl2*), and fibrillin-1 (*Fbn1*) exhibited the most significant colocalization with senescent endothelial cells based on gene expression correlation scores (Figure 1M). Specifically, TGM2 and LOXL2 are ECM-modifying enzymes, whereas FBN1 is a major structural glycoprotein of the matrix. Based on its structural role and significant colocalization, we postulated that FBN1 could alter the peritubular niche and promote endothelial cell senescence, consistent with prior reports of its involvement in endothelial injury 24. To evaluate this, we first confirmed that the spatial expression profile of *Fbn1* showed a high degree of concordance with the cortical distribution of senescent endothelial cells (Figure 1N). Further validation via immunofluorescence staining demonstrated significant vascular endothelial rarefaction in renal fibrosis models induced by UUO, UIRI, and AAN. Importantly, the rarefied endothelial cells displayed γ-H2AX positivity, confirming the presence of senescence features. More importantly, senescent endothelial cells were markedly enriched in the peritubular space, a region where tubules exhibited strong FBN1 expression (Figure 1O). Based on these lines of evidence, we hypothesize that FBN1 may establish a pro-senescent microenvironment that critically exacerbates endothelial senescence during renal fibrosis.

### FBN1 induces endothelial cell senescence *in vitro*

**Figure 2:**
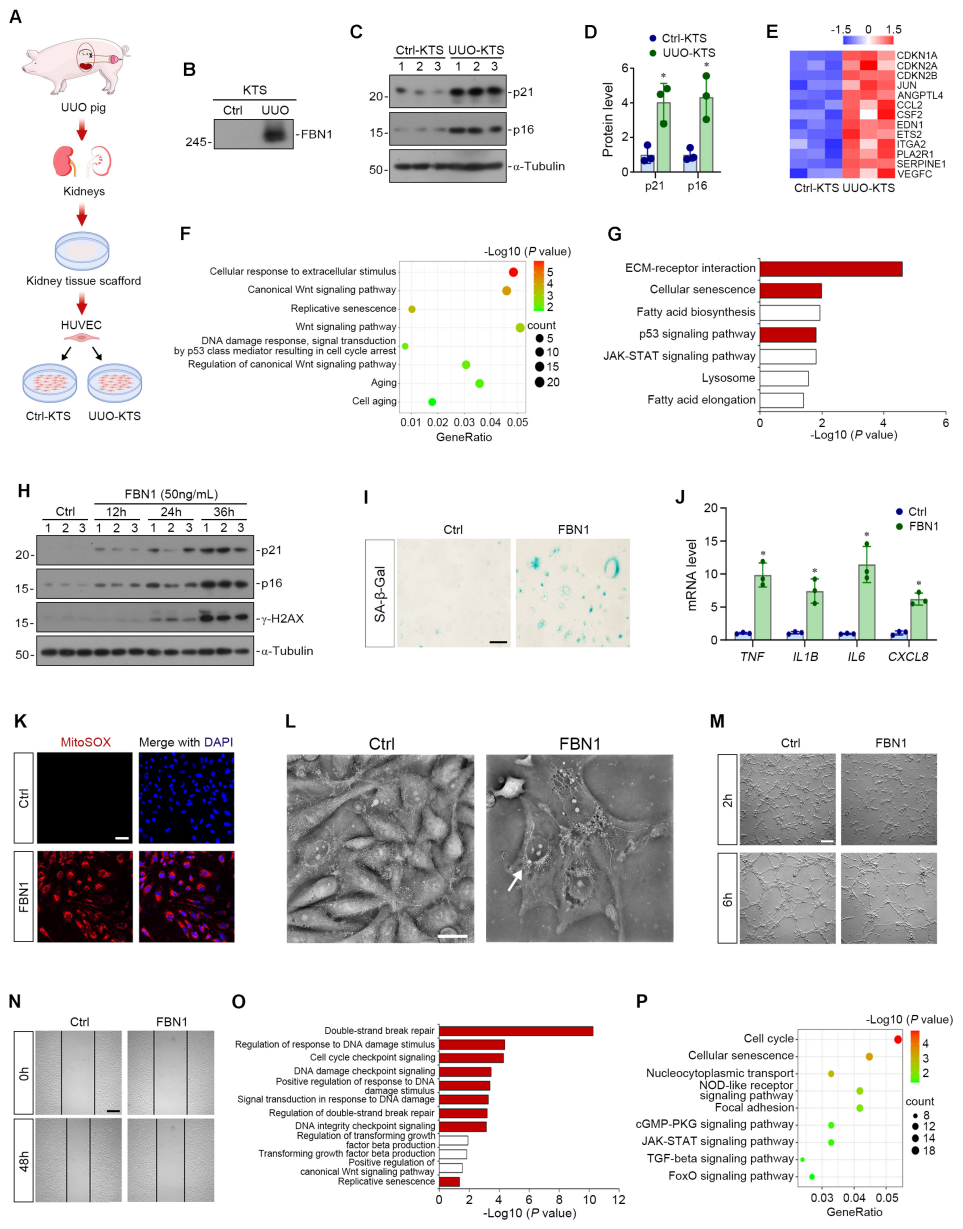
** FBN1 induces endothelial cell senescence *in vitro*. (A)** The experimental protocol of the study. Kidney tissue scaffolds (KTS) were prepared from pig kidneys of control (Ctrl) and UUO groups, followed by culture of human umbilical vein endothelial cells (HUVECs) on the scaffolds. **(B)** Western blot shows the expression of FBN1 protein in Ctrl-KTS and UUO-KTS. **(C-D)** Representative Western blot (C) and quantitative data (D) show the expression of p21 and p16 proteins in HUVECs cultured on different KTS. *P < 0.05 versus Ctrl-KTS (n = 3). **(E)** The heatmap shows the mRNA expression levels of a panel of senescence-associated genes in HUVECs cultured on Ctrl- or UUO-KTS. **(F)** GO enrichment analysis reveals a set of significantly enriched biological pathways. **(G)** KEGG enrichment analysis identifies several enriched pathways as indicated. **(H)** Representative Western blot analyses show the expression of p21, p16 and γ-H2AX proteins in HUVECs after FBN1 stimulation at different time points. **(I)** Representative micrographs show the positive senescence-associated β-galactosidase (SA-β-gal) staining in different groups as indicated. Scale bar, 50 μm.** (J)** Quantitative real-time polymerase chain reaction (qPCR) analyses of mRNA expression levels of *TNF*, *IL1B*, *IL6*, and *CXCL8* in different groups of HUVECs. *P < 0.05 versus Ctrl (n = 3). **(K)** Representative micrographs show MitoSOX staining in different groups. Scale bar, 50 μm.** (L)** Label-free live-cell morphology imaging revealing characteristic senescent features in FBN1-treated cells. Arrows indicate fragmented mitochondria. Scale bar, 30 μm.** (M)** Representative micrographs show the formation of vascular networks at 2 h and 6 h in different groups. Scale bar, 200 μm. **(N)** Representative micrographs show the wound area at 0 h and 48 h in different groups. Scale bar, 400 μm.** (O)** GO enrichment analysis reveals a set of significantly enriched biological pathways. **(P)** KEGG enrichment analysis identifies several enriched pathways as indicated.

To elucidate the role of FBN1 in endothelial cell senescence, we first established a UUO model in Tibetan minipigs and prepared decellularized KTS. These scaffolds, enriched with FBN1, serve as a pathomimetic *in vitro* system for investigating the functional contribution of FBN1 to endothelial senescence. HUVECs were then inoculated on KTS derived from control pigs and UUO pigs (Figure 2A). Compared with Ctrl-KTS, UUO-KTS showed abundant FBN1 protein (Figure 2B). In addition, HUVECs inoculated on UUO-KTS exhibited high expression levels of p21 and p16 proteins (Figure 2C-D). Furthermore, RNA sequencing (RNA-seq) showed that a panel of senescence-associated genes was upregulated in HUVECs cultured on UUO-KTS (Figure 2E). GO enrichment analysis revealed that HUVECs on UUO-KTS showed significant enrichment in several key signaling pathways, such as the cellular response to extracellular stimulus, Wnt signaling pathway, replicative senescence, and DNA damage response (Figure 2F). Furthermore, KEGG enrichment analysis confirmed marked enrichment of the ECM-receptor interaction, cellular senescence, and p53 signaling pathways in HUVECs cultured on UUO-KTS (Figure 2G). These results demonstrate that the FBN1-enriched microenvironment drives endothelial senescence during kidney fibrosis.

To directly confirm the role of FBN1 in promoting endothelial cell senescence, HUVECs were treated with increasing concentrations of human recombinant FBN1 protein. Given that 50 ng/mL FBN1 robustly induced senescence in dose-response experiments (Figure S1A-B), this concentration was selected for subsequent studies. After treatment with 50 ng/mL FBN1 for varying durations, Western blot analyses revealed that FBN1 significantly upregulated p16, p21, and γ-H2AX proteins in a time-dependent manner (Figure 2H, Figure S1C), with consistent results from senescence-associated β-galactosidase (SA-β-Gal) staining (Figure 2I, Figure S1D). qPCR showed that FBN1 significantly upregulated the mRNA expression of *TNF*, *IL1B*, *IL6*, and *CXCL8* in HUVECs, indicating that FBN1 promoted the senescence-associated secretory phenotype (SASP) in endothelial cells (Figure 2J). Given enhanced oxidative stress in senescent cells, we performed MitoSOX staining to quantify intracellular reactive oxygen species (ROS) levels. Results demonstrated that FBN1 markedly elevated ROS levels in HUVECs, thereby confirming its ability to induce endothelial cell senescence (Figure 2K, Figure S1E). Furthermore, we performed label-free imaging via optical diffraction tomography (ODT) to assess cellular morphology. As shown in Figure 2L, compared with control cells, HUVECs treated with FBN1 for 36 h exhibited a series of senescence-associated morphological changes, including flattened cell bodies, cytoplasmic vacuolization, nuclear hypertrophy, and fragmented mitochondria aggregated in the perinuclear region.

We then conducted tube formation assays to evaluate angiogenic potential. At 2 h of incubation, control cells were densely distributed and initiated tube-like structure formation, whereas FBN1-treated cells were sparse and scattered. At 6 h, control cells formed well-developed vascular networks characterized by clear lumens, abundant branches, and closed loops. By contrast, FBN1-treated cells remained dispersed, failing to form closed lumens with few branches and fragmented networks (Figure 2M, Figure S1F-H). These observations indicate that FBN1 treatment rapidly impairs endothelial cell viability. Next, a scratch wound assay was performed to assess cell migratory capacity. Initial scratch width was consistent between groups at 0 h. After 48 h, control cells migrated prominently into the scratch area, whereas FBN1-treated cells exhibited drastically impaired migration (Figure 2N, Figure S1I), suggesting FBN1 inhibits endothelial migration. RNA-seq further demonstrated that FBN1 treatment induced a significant alteration in the gene expression profile of HUVECs compared with the control group (Figure S1J). GO enrichment analysis indicated significant enrichment of DNA damage, cell cycle regulation, Wnt signaling, and cellular senescence pathways in FBN1-treated cells (Figure 2O). Consistently, KEGG analysis confirmed enrichment in cell cycle and cellular senescence pathways in these cells (Figure 2P). To further assess the cell-type specificity of FBN1-induced senescence, we performed *in vitro* experiments using HK-2 cells, a human renal tubular epithelial cell line. Notably, no significant induction of senescence was observed in HK-2 cells following FBN1 treatment, indicating that the pro-senescent effect of FBN1 is preferentially directed toward endothelial cells (Figure S1K-L). Collectively, these integrated molecular, morphological, functional, and transcriptomic analyses demonstrate that FBN1 acts as a master regulator that induces endothelial cell senescence *in vitro*.

### ET-1/β-catenin axis conveys FBN1-derived pro-senescent signaling

**Figure 3:**
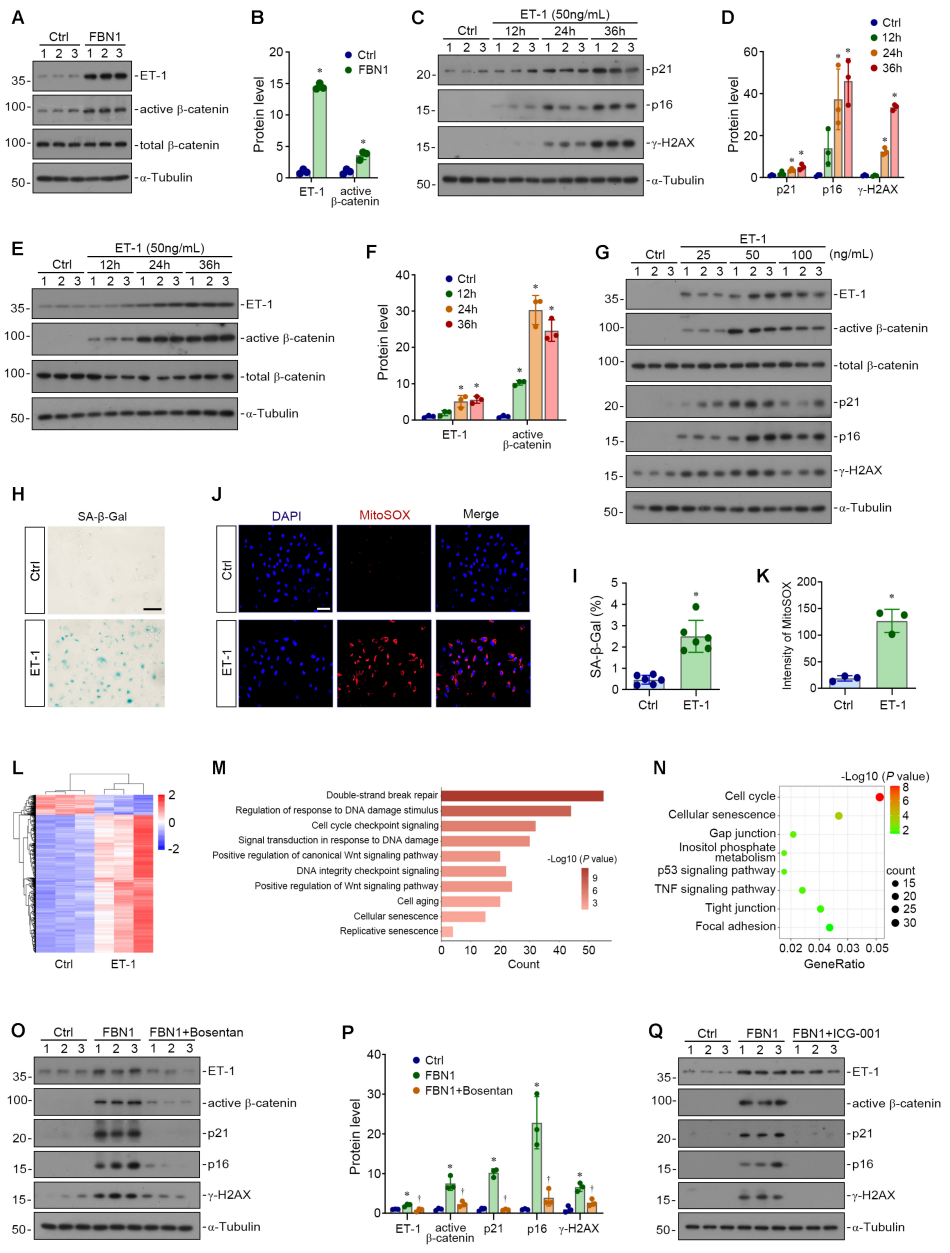
** ET-1/β-catenin axis conveys FBN1-derived pro-senescent signaling. (A-B)** Representative Western blot (A) and quantitative data (B) show the protein expression levels of ET-1, active β-catenin and total β-catenin. *P < 0.05 versus Ctrl (n = 3). **(C-D)** Representative Western blot (C) and quantitative data (D) show the expression of p21, p16 and γ-H2AX proteins in Ctrl and ET-1-treated cells at different time points as indicated (n = 3). **(E-F)** Representative Western blot (E) and quantitative data (F) show the expression of ET-1, active β-catenin and total β-catenin proteins in Ctrl and ET-1-treated cells at different time points as indicated. *P < 0.05 versus Ctrl (n = 3). **(G)** Representative Western blot analyses show the expression of ET-1, active β-catenin, total β-catenin, p21, p16 and γ-H2AX proteins in HUVECs treated with different concentrations of ET-1. **(H-I)** Representative micrographs (H) and quantitative data (I) show the SA-β-gal positive cells in Ctrl and ET-1-treated groups. *P < 0.05 versus Ctrl (n = 6). Scale bar, 50 μm.** (J-K)** Representative micrographs (J) and quantitative data (K) show MitoSOX staining in Ctrl and ET-1-treated groups. *P < 0.05 versus Ctrl (n = 3). Scale bar, 50 μm.** (L)** The heatmap shows the gene expression profiles in Ctrl and ET-1-treated cells. **(M)** GO enrichment analysis reveals a set of significantly enriched biological pathways. **(N)** KEGG enrichment analysis identifies several enriched pathways as indicated. **(O-P)** Representative Western blot (O) and quantitative data (P) show the expression of ET-1, active β-catenin, total β-catenin, p21, p16 and γ-H2AX proteins in different groups as indicated. *P < 0.05 versus Ctrl, †*P* < 0.05 versus FBN1 (n = 3). **(Q)** Representative Western blot analyses show the expression of ET-1, active β-catenin, total β-catenin, p21, p16 and γ-H2AX proteins in different groups as indicated.

Having established that FBN1 directly induces endothelial cell senescence, we next turned to investigate its underlying molecular mechanisms. As a critical factor in endothelial injury response, endothelin-1 (ET-1) can activate the Wnt/β-catenin pathway, whose dysregulation is closely associated with cellular senescence. Of note, our single-cell analysis showed marked activation of the β-catenin pathway in senescent versus homeostatic endothelial cells (Figure S2A). Therefore, we hypothesized that FBN1 might trigger senescence by inducing ET-1 expression and activating the β-catenin pathway. First, we found that FBN1 directly induced ET-1 protein expression and β-catenin activation in HUVECs (Figure 3A-B). Further experiments revealed that ET-1 induced HUVEC senescence and β-catenin activation in a time-dependent manner (Figure 3C-F). Additionally, ET-1 could activate β-catenin and induce endothelial cell senescence in a dose-dependent manner (Figure 3G, Figure S2B). Moreover, results from SA-β-gal staining and MitoSOX staining also indicated that ET-1 directly induced endothelial cell senescence and oxidative stress (Figure 3H-K).

We then performed RNA-seq to investigate the gene expression profile of endothelial cells upon ET-1 stimulation. The results showed that ET-1 stimulation caused a significant alteration in gene expression compared with the control group (Figure 3L). Further GO enrichment analysis demonstrated that pathways related to DNA damage, cell cycle regulation, Wnt signaling pathway, and cellular senescence were significantly enriched in ET-1-treated cells (Figure 3M). KEGG enrichment analysis also indicated the significant enrichment of pathways associated with cell cycle, cellular senescence, p53 signaling pathway, and TNF signaling pathway (Figure 3N). These results further suggest that ET-1 can induce endothelial cell senescence *in vitro*.

To further investigate the contribution of ET-1 signaling specifically, we used Bosentan, an ET-1 receptor antagonist, and assessed its effects on FBN1-triggered cellular senescence. We found that inhibition of ET-1 receptors significantly suppressed the FBN1-induced activation of the ET-1/β-catenin pathway, as well as the expression of senescence-associated proteins and SASP-related proteins (Figure 3O-P, Figure S2C-D). Furthermore, ICG-001, an inhibitor of β-catenin-mediated transcription, markedly blocked FBN1-induced β-catenin activation and alleviated endothelial cell senescence (Figure 3Q, Figure S2E). Collectively, our findings coherently define a linear mechanism whereby FBN1 drives endothelial senescence through ET-1 induction and subsequent β-catenin activation. The ability of both ET-1 antagonism and β-catenin inhibition to intercept this cascade provides robust functional validation. Thus, these results outline a pathogenic sequence linking matrix-derived FBN1 to cellular senescence and highlight potential actionable signaling targets within this network.

### ZEB1 mediates FBN1-induced ET-1/β-catenin activation in endothelial senescence

**Figure 4:**
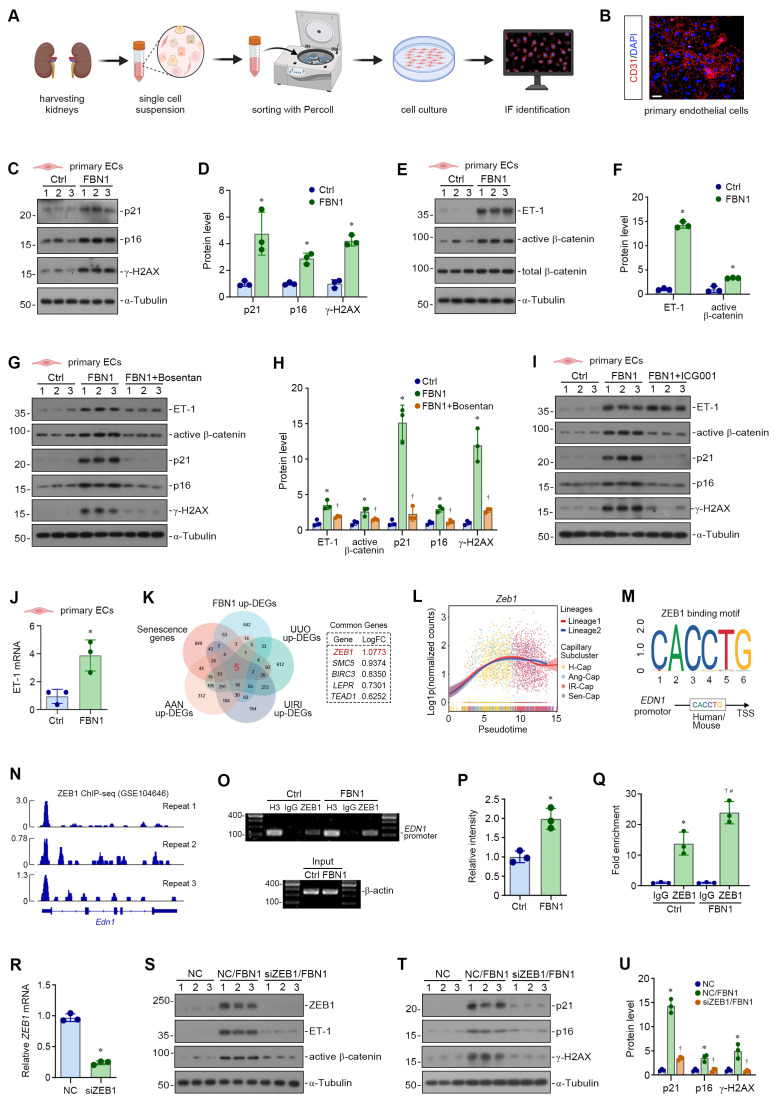
** ZEB1 mediates FBN1-induced ET-1/β-catenin activation in endothelial senescence. (A)** Diagram shows the experimental protocol for isolating renal primary endothelial cells. **(B)** Immunofluorescence staining for CD31. Scale bar, 50 μm.** (C-D)** Representative Western blot (C) and quantitative data (D) show the expression of p21, p16 and γ-H2AX proteins in primary endothelial cells from different groups (n = 3). **(E-F)** Representative Western blot (E) and quantitative data (F) show the expression of ET-1, active β-catenin and total β-catenin proteins in primary endothelial cells from different groups. *P < 0.05 versus Ctrl (n = 3).** (G-H)** Representative Western blot (G) and quantitative data (H) show the expression of ET-1, active β-catenin, p21, p16 and γ-H2AX proteins in different groups as indicated. *P < 0.05 versus Ctrl, †*P* < 0.05 versus FBN1 (n = 3). **(I)** Representative Western blot analyses show the expression of ET-1, active β-catenin, p21, p16 and γ-H2AX proteins in different groups as indicated. **(J)** qPCR analyses of ET-1 mRNA expression in different groups of primary endothelial cells. *P < 0.05 versus Ctrl (n = 3). **(K)** The venn diagram shows the intersection among upregulated differentially expressed genes (DEGs) in FBN1-treated cells, upregulated DEGs from peritubular capillary endothelial cells in UIRI, UUO and AAN models, and a comprehensive senescence gene set. **(L)** Dynamic expression of *Zeb1* along pseudotime trajectories (Lineage 1 and Lineage 2). Cells are colored by capillary endothelial subtype, and trajectory curves illustrate functional state transitions. Differences between lineages highlight distinct temporal expression patterns of *Zeb1*. **(M)** Bioinformatic analysis using the JASPAR database revealed that ZEB1 can bind to high-confidence putative sites in the promoter region of the human *EDN1* or mouse *Edn1* gene (threshold: 95%). **(N)** ZEB1-binding peak at the *Edn1* transcription start site identified via public ChIP-seq dataset (GSE104646).** (O)** Representative ChIP assay demonstrates ZEB1 binding to the *EDN1* promoter region, an interaction that is markedly potentiated by FBN1 treatment. **(P)** Semiquantitative analysis of ChIP-PCR products from samples immunoprecipitated with ZEB1 antibody, comparing Ctrl and FBN1-treated groups. *P < 0.05 versus Ctrl (n = 3). **(Q)** Quantitative ChIP-qPCR results confirm enhanced ZEB1 enrichment at the *EDN1* promoter following FBN1 stimulation. *P < 0.05 versus Ctrl-IgG, †*P* < 0.05 versus FBN1-IgG, #*P* < 0.05 versus Ctrl-ZEB1 (n = 3). **(R)** qPCR analyses show the knockdown efficiency of *ZEB1* mRNA after transfection with small interfering RNA targeting *ZEB1* (siZEB1). *P < 0.05 versus NC (n = 3). **(S)** Representative Western blot analyses show the expression of ZEB1, ET-1 and active β-catenin proteins in different groups as indicated. **(T-U)** Representative Western blot (T) and quantitative data (U) show the expression of p21, p16 and γ-H2AX proteins in different groups as indicated. *P < 0.05 versus NC, †*P* < 0.05 versus NC/FBN1 (n = 3).

To better model the response of renal endothelial cells to FBN1, we isolated primary renal endothelial cells. Figure 4A illustrates the experimental workflow. CD31 immunofluorescence staining confirmed successful enrichment of these cells (Figure 4B). Subsequently, we treated primary renal endothelial cells with recombinant FBN1 protein and found that the expression of p21, p16, and γ-H2AX proteins was significantly induced (Figure 4C-D). In addition, FBN1 significantly induced the activation of the ET-1/β-catenin pathway (Figure 4E-F). Moreover, blockade of ET-1 with Bosentan suppressed FBN1-induced ET-1/β-catenin pathway activation and cellular senescence in primary renal endothelial cells (Figure 4G-H). Similarly, β-catenin inhibition via ICG-001 attenuated FBN1-triggered β-catenin activation and alleviated endothelial senescence (Figure 4I, Figure S3A). Collectively, these results confirm in primary renal endothelial cells that FBN1 promotes cellular senescence via the ET-1/β-catenin pathway, and that this effect is mitigated by either ET-1 receptor antagonism or direct β-catenin inhibition.

To clarify the mechanism by which FBN1 upregulates ET-1, we assessed ET-1 mRNA level in control and FBN1-treated primary renal endothelial cells by qPCR. As shown in Figure 4J, FBN1 promoted the upregulation of ET-1 mRNA levels, suggesting that FBN1 might induce ET-1 protein expression via transcriptional regulation. Therefore, in order to identify the regulatory factors involved in ET-1 mRNA upregulation, we performed an integrated analysis by intersecting the following datasets: upregulated DEGs in FBN1-stimulated endothelial cells, upregulated DEGs from peritubular capillary endothelial cells in UIRI, UUO, AAN models, and a comprehensive senescence gene set—defined as the union of the GO term “cellular senescence” (GO:0090398) together with three well-curated, published senescence gene sets 53-55. This integrated analysis identified five conserved, FBN1-induced, senescence-associated genes in the various CKD models: *ZEB1*, *SMC5*, *BIRC3*, *LEPR*, and *TEAD1*. Among these, the transcription factor *ZEB1* exhibited the greatest upregulation in FBN1-stimulated endothelial cells (Figure 4K). We also found that *Zeb1* was significantly overexpressed in peritubular capillary endothelial cells from three CKD models, including UUO, UIRI, and AAN (Figure S3B). Pseudotime trajectory analysis further revealed that *Zeb1* expression was progressively upregulated as homeostatic endothelial cells advanced along lineage toward the senescent state (Figure 4L). Moreover, *ZEB1* was the only candidate gene for which bioinformatic analysis verified a high-confidence binding motif in the *EDN1* promoter region (Figure 4M). We therefore hypothesized that ZEB1 mediates FBN1-induced ET-1 upregulation via direct transcriptional modulation. Consistent with this hypothesis, inspection of a public ZEB1 ChIP-seq dataset (GSE104646) demonstrated a sharp ZEB1-binding peak at the transcription start site of the *Edn1* gene (Figure 4N). Moreover, we performed ChIP assays in our model to experimentally validate the specific interaction between ZEB1 and the *EDN1* promoter (Figure 4O-Q). ChIP assays provided direct evidence for the specific binding of ZEB1 to the *EDN1* promoter in control cells and further showed that FBN1 treatment robustly augmented this association. This not only confirms the role of ZEB1 in regulating *EDN1* transcription but also reveals that FBN1 enhances *EDN1* expression by promoting ZEB1 occupancy at the promoter.

To further confirm the role of ZEB1 in FBN1-induced endothelial cell senescence, we transfected HUVECs with small interfering RNA targeting *ZEB1* (siZEB1). The approximately 80% knockdown of *ZEB1* mRNA confirmed the transfection efficiency (Figure 4R). Western blot analyses showed that *ZEB1* silencing inhibited FBN1-induced ZEB1 expression, suppressed ET-1/β-catenin pathway activation (Figure 4S, Figure S3C), and attenuated cellular senescence (Figure 4T-U). Taken together, our results demonstrate that FBN1 upregulates the transcription factor ZEB1, which in turn promotes ET-1 expression via direct promoter binding. These findings establish the ZEB1/ET-1/β-catenin axis as a critical signaling cascade responsible for FBN1-induced endothelial senescence.

### FBN1 aggravates renal senescence in unilateral ureteral obstruction model

**Figure 5:**
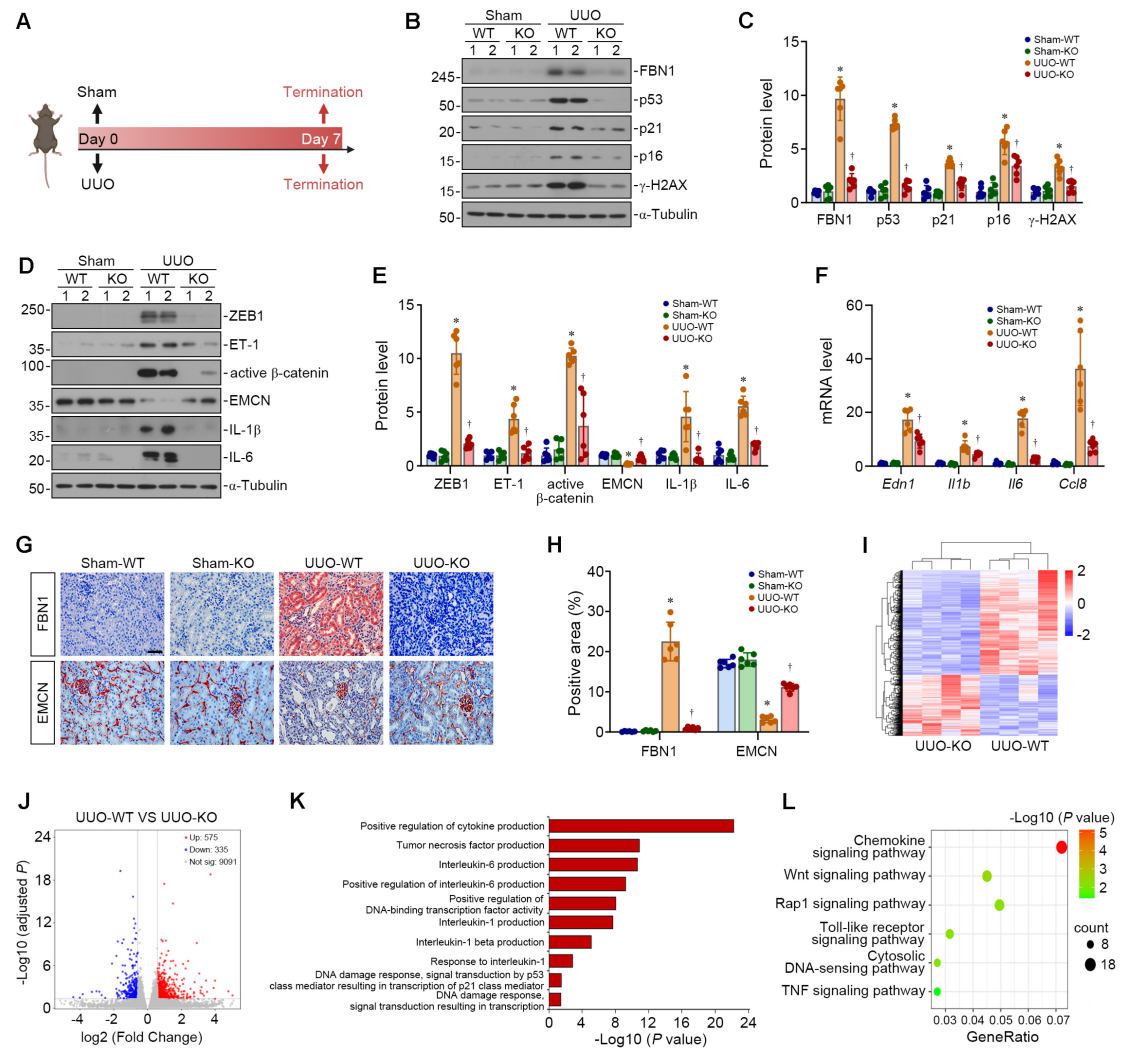
** FBN1 aggravates renal senescence in unilateral ureteral obstruction model. (A)** The experimental protocol of UUO model. **(B-C)** Representative Western blot (B) and quantitative data (C) show the renal expression of FBN1, p53, p21, p16 and γ-H2AX proteins in different groups as indicated (n = 6). **(D-E)** Representative Western blot (D) and quantitative data (E) show the renal expression of ZEB1, ET-1, active β-catenin, EMCN, IL-1β and IL-6 proteins in different groups as indicated. *P < 0.05 versus Sham-WT, †*P* < 0.05 versus UUO-WT (n = 6). **(F)** qPCR analyses show the renal mRNA expression levels of *Edn1*, *Il1b*, *Il6*, and *Ccl8*. *P < 0.05 versus Sham-WT, †*P* < 0.05 versus UUO-WT (n = 6). **(G)** Representative micrographs show the renal expression and localization of FBN1 and EMCN in different groups by immunohistochemical staining. Scale bar, 50 μm.** (H)** Quantitative analyses of immunohistochemical staining for FBN1 and EMCN. At least 10 randomly selected microscopic fields were assessed, and the results were averaged for each kidney. *P < 0.05 versus Sham-WT, †*P* < 0.05 versus UUO-WT (n = 6). **(I)** The heatmap shows the gene expression profiles in UUO-WT and UUO-KO. **(J)** Volcano plot shows the DEGs in UUO-WT compared with UUO-KO. **(K)** GO enrichment analysis reveals a set of significantly enriched biological pathways. **(L)** KEGG enrichment analysis identifies several enriched pathways as indicated.

To investigate the role of FBN1 in renal senescence *in vivo*, we generated tubule-specific *Fbn1* knockout (KO) mice. As shown in Figure 5A, we established the UUO model in wild-type (WT) and KO mice. Quantitative PCR analysis of whole-kidney tissue validated the efficiency of transcript ablation (Figure S4A). Western blot analyses demonstrated that the expression of FBN1 and senescence-associated proteins such as p53, p21, p16, and γ-H2AX was significantly upregulated in UUO-WT mice, whereas their expression was markedly suppressed in UUO-KO mice (Figure 5B-C). Furthermore, in UUO-WT mice, the ZEB1/ET-1/β-catenin pathway was activated, endothelial marker EMCN expression was downregulated, and the expression of SASP-related proteins IL-1β and IL-6 was upregulated. In contrast, in UUO-KO mice, the activation of the ET-1/β-catenin pathway was inhibited, the expression of EMCN was restored, and the expression of IL-1β and IL-6 was suppressed (Figure 5D-E). Consistently, the renal mRNA levels of *Edn1*, *Il1b*, *Il6*, and *Ccl8* were elevated in the obstructed kidneys of WT mice, which were largely abolished by deletion of *Fbn1* in the KO mice (Figure 5F). Immunohistochemical staining (IHC) for FBN1 showed high FBN1 expression in renal tubules of UUO-WT mice, whereas FBN1 expression in renal tubules was markedly reduced in UUO-KO mice. IHC staining for EMCN revealed capillary rarefaction in UUO-WT mice, which was ameliorated by *Fbn1* knockout (Figure 5G-H).

We then employed the RNA-seq approach to investigate the gene expression profile of kidneys in different groups. The mRNA clustering showed significant differences in the gene expression between UUO-WT mice and UUO-KO mice (Figure 5I). Volcano plot analysis identified 575 significantly upregulated DEGs and 335 significantly downregulated DEGs in UUO-WT mice compared with UUO-KO mice (Figure 5J). GO enrichment analysis indicated that SASP-related pathways and DNA damage-related pathways were significantly enriched in UUO-WT kidneys (Figure 5K). Similarly, KEGG enrichment analysis showed that Chemokine signaling pathway and Wnt signaling pathway were markedly enriched in UUO-WT kidneys (Figure 5L). Together, these results suggest that *Fbn1* knockout significantly alleviates DNA damage and SASP secretion in UUO mice, thereby ameliorating renal senescence.

### FBN1 drives endothelial senescence and capillary rarefaction in UUO mice

**Figure 6:**
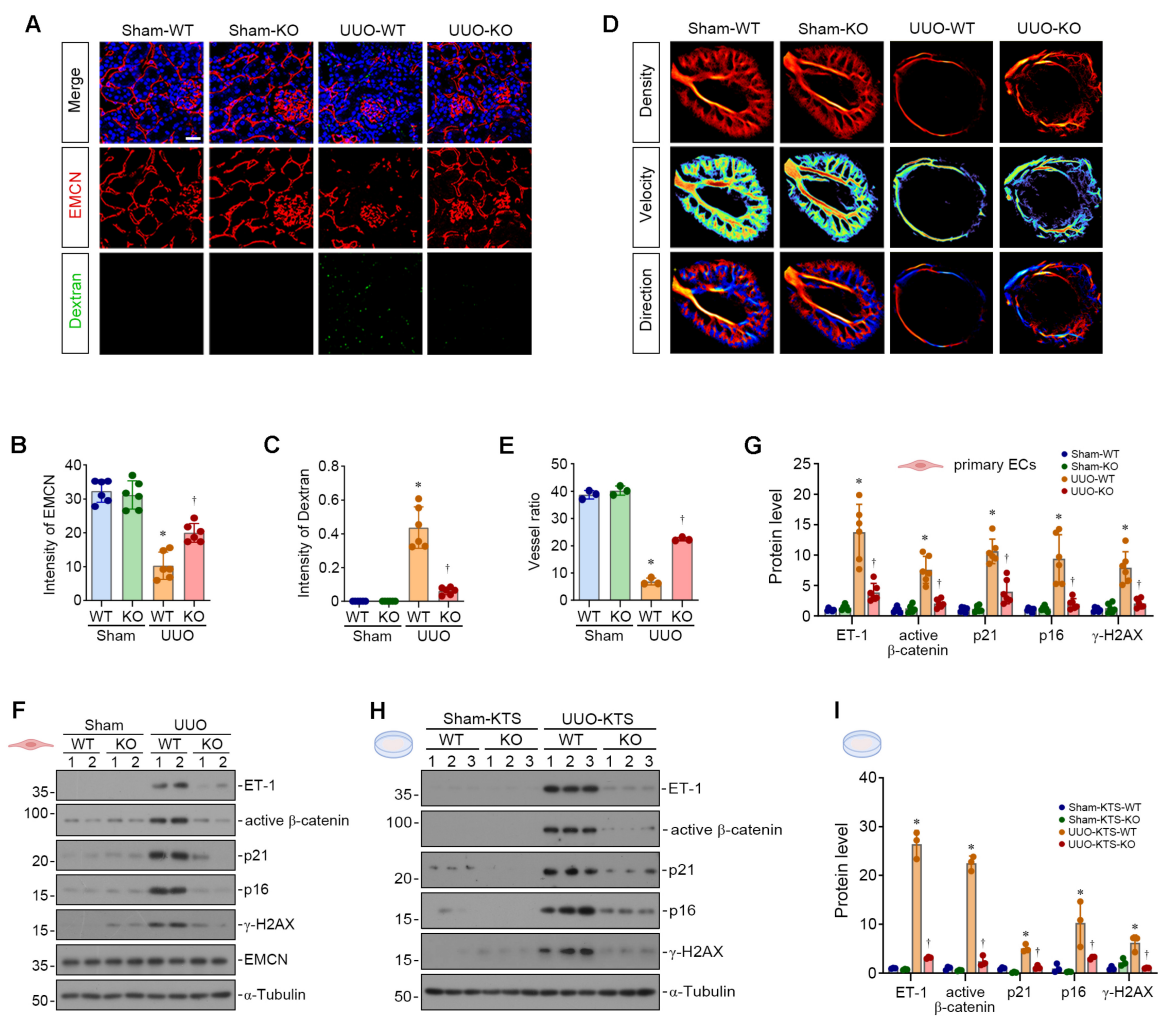
** FBN1 drives endothelial senescence and capillary rarefaction in UUO mice. (A-C)** Vascular permeability assay. The mice in different groups were injected with FITC-dextran through tail vein. Representative micrographs (A) show the immunofluorescence staining of EMCN and the fluorescence signals of FITC-dextran. Quantitative data (B-C) show the fluorescence intensity of EMCN and FITC-dextran signals. *P < 0.05 versus Sham-WT, †*P* < 0.05 versus UUO-WT (n = 6). Scale bar, 25 μm.** (D-E)** Representative micrographs (D) show the ultrasound of renal microvasculature in different groups as indicated. Ultra-resolution microscopy (URM) imaging was performed to visualize the functional maps of microvascular density, blood flow velocity, and flow direction. Histogram (E) shows the vessel ratio in different groups as indicated. *P < 0.05 versus Sham-WT, †*P* < 0.05 versus UUO-WT (n = 3). **(F-G)** Representative Western blot (F) and quantitative data (G) show the expression of ET-1, active β-catenin, p21, p16, γ-H2AX and EMCN proteins in different groups of renal primary endothelial cells. *P < 0.05 versus Sham-WT, †*P* < 0.05 versus UUO-WT (n = 6). **(H-I)** HUVECs were inoculated on different KTS as indicated. Representative Western blot (H) and quantitative data (I) show the expression of ET-1, active β-catenin, p21, p16 and γ-H2AX proteins in different groups as indicated. *P < 0.05 versus Sham-KTS-WT, †*P* < 0.05 versus UUO-KTS-WT (n = 3).

We then clarified the effect of FBN1 on renal vascular endothelial cell function by examining FITC-dextran leakage. Scattered fluorescent signals were observed in the renal interstitium of UUO-WT mice, whereas such signals were significantly attenuated in UUO-KO mice, indicating that the depletion of *Fbn1* markedly improved vascular endothelial permeability in UUO mice (Figure 6A-C). Next, ultra-resolution ultrasound microscopy was performed to further evaluate renal microvascular hemodynamics across different groups. We found that renal microvessels in sham-operated kidneys appeared clear with abundant terminals, indicating dense microvascular networks with abundant blood supply. However, in the obstructed kidneys in WT mice, only the main renal artery was visible, with sparse and indistinct microvessels, accompanied by significantly decreased vascular ratio, complexity, and perfusion index. In contrast, obstructed kidneys from *Fbn1* knockout mice exhibited clearly visible microvessels with relatively abundant terminals, and these three indices were significantly restored (Figure 6D-E, Figure S4B-C). These results indicate that knockout of *Fbn1* can protect the kidney microvasculature and kidney blood flow.

To precisely characterize the effect of FBN1 on endothelial cells in UUO mice, we isolated primary renal endothelial cells from different groups of mice. We found that primary renal endothelial cells from UUO-WT mice exhibited significant activation of the ET-1/β-catenin pathway and marked upregulation of senescence-associated proteins compared with those from Sham-WT mice. In contrast, the ET-1/β-catenin pathway was inhibited and the expression of these senescence-associated proteins was suppressed in primary renal endothelial cells from UUO-KO mice (Figure 6F-G). Next, we explored whether FBN1 establishes a pro-senescent microenvironment for endothelial cells in UUO mice. We prepared decellularized KTS from the above four groups of mice and inoculated HUVECs onto these scaffolds. Endothelial cells on UUO-WT KTS exhibited robust ET-1/β-catenin pathway activation and elevated p21, p16, and γ-H2AX levels compared with those on Sham-WT KTS. Conversely, these responses were suppressed in endothelial cells on UUO-KO KTS (Figure 6H-I). These findings indicate that FBN1 promotes endothelial cell senescence and capillary rarefaction in UUO mice by establishing a specific pro-senescent microenvironment.

### FBN1 drives post-ischemic microvascular degeneration via senescence acceleration

**Figure 7:**
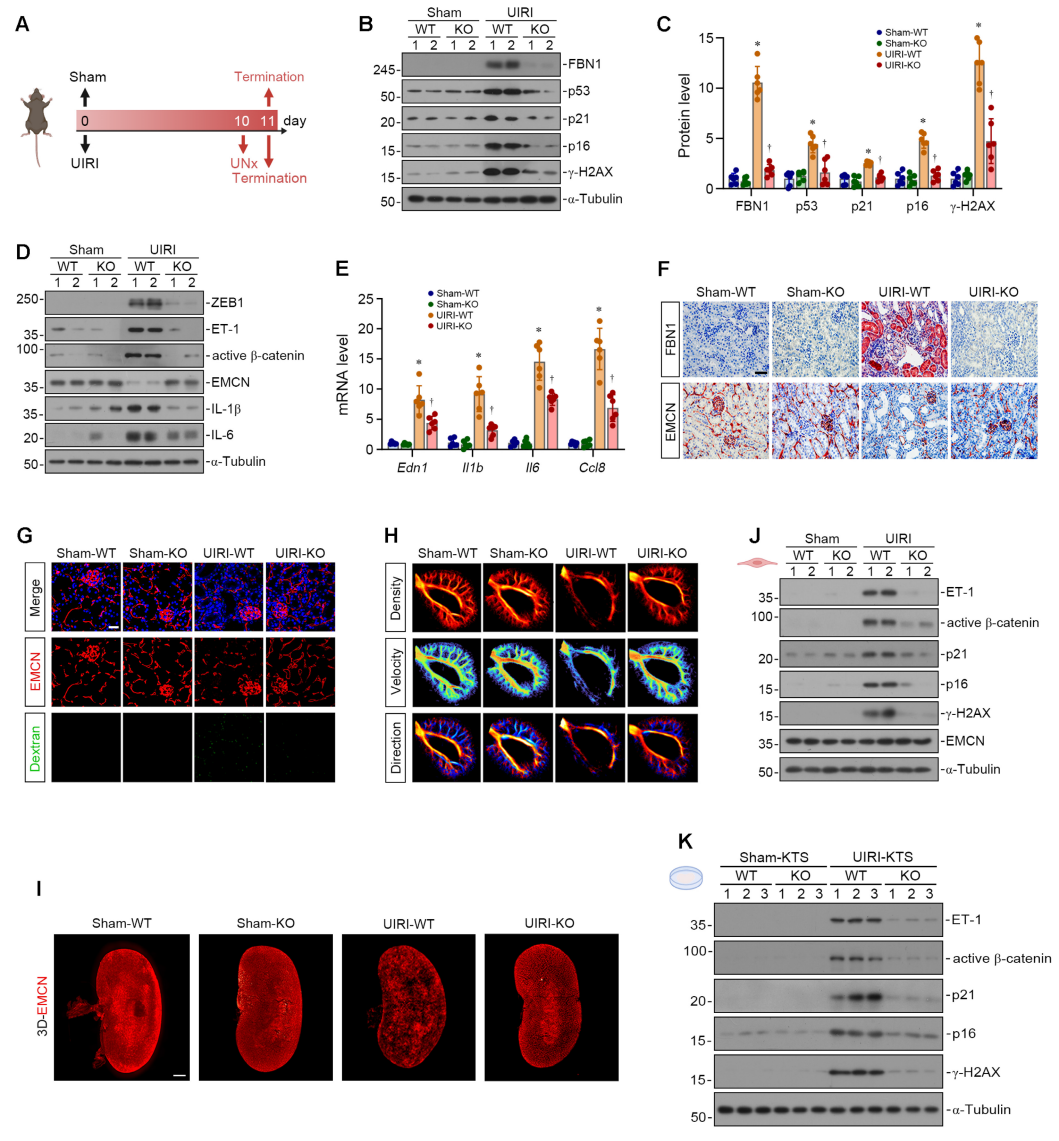
** FBN1 drives post-ischemic microvascular degeneration via senescence acceleration. (A)** The experimental protocol of UIRI model. **(B-C)** Representative Western blot (B) and quantitative data (C) show the renal expression of FBN1, p53, p21, p16 and γ-H2AX proteins in different groups as indicated. *P < 0.05 versus Sham-WT, †*P* < 0.05 versus UIRI-WT (n = 6). **(D)** Representative Western blot analyses show the renal expression of ZEB1, ET-1, active β-catenin, EMCN, IL-1β and IL-6 proteins in different groups as indicated. **(E)** qPCR analyses show the renal mRNA expression levels of *Edn1*, *Il1b*, *Il6*, and *Ccl8*. *P < 0.05 versus Sham-WT, †*P* < 0.05 versus UIRI-WT (n = 6). **(F)** Representative micrographs show the renal expression and localization of FBN1 and EMCN in different groups by immunohistochemical staining. Scale bar, 50 μm.** (G)** Vascular permeability assay. Representative micrographs show the immunofluorescence staining of EMCN and the fluorescence signals of FITC-dextran. Scale bar, 25 μm.** (H)** Representative micrographs show the ultrasound of renal microvasculature in different groups as indicated. **(I)** Representative micrographs show the 3D microvascular networks in mouse kidneys from different groups, obtained via the tissue clearing technique. Vasculature was labeled with EMCN. Scale bar, 1 mm.** (J)** Representative Western blot analyses show the expression of ET-1, active β-catenin, p21, p16, γ-H2AX and EMCN proteins in different groups of renal primary endothelial cells. **(K)** HUVECs were inoculated on different KTS as indicated. Representative Western blot analyses show the expression of ET-1, active β-catenin, p21, p16 and γ-H2AX proteins in different groups.

To further explore the role of FBN1 in CKD, we established the UIRI model in WT and KO mice, as illustrated in Figure 7A. First, we evaluated the effect of *Fbn1* knockout on renal function in UIRI mice. As shown in Figure S5A-B, *Fbn1* knockout reduced serum creatinine (Scr) and blood urea nitrogen (BUN) levels in UIRI mice. Western blot analyses demonstrated that knockout of *Fbn1* alleviated renal senescence in UIRI mice (Figure 7B-C). This was accompanied by inhibition of the ZEB1/ET-1/β-catenin pathway, restoration of EMCN expression, and suppression of SASP-related proteins (Figure 7D, Figure S5C). Furthermore, FBN1 knockout reduced the renal mRNA levels of *Edn1*, *Il1b*, *Il6*, and *Ccl8* in UIRI mice (Figure 7E). IHC staining revealed high FBN1 expression in renal tubules of UIRI-WT mice. Meanwhile, UIRI-WT mice exhibited renal capillary rarefaction, a phenotype that was significantly ameliorated by *Fbn1* knockout (Figure 7F, Figure S5D).

We then performed a FITC-dextran leakage assay to assess the impact of *Fbn1* knockout on vascular endothelial function in UIRI mice. *Fbn1* knockout markedly attenuated renal interstitial fluorescent signals in UIRI mice, indicating improved vascular endothelial permeability (Figure 7G, Figure S5E-F). Ultra-resolution ultrasound microscopy assessments demonstrated that UIRI-KO mice exhibited well-visualized microvessels with relatively abundant peripheral branches, comparable to those in sham groups. Furthermore, the vascular ratio, complexity, and perfusion index in UIRI-KO mice were markedly restored compared with UIRI-WT mice (Figure 7H, Figure S5G-I). Next, we applied tissue-clearing combined with immunofluorescent labeling for EMCN to enable high-resolution three-dimensional visualization of the intrarenal vasculature. Sham group kidneys displayed abundant blood vessels with strong fluorescent signals. UIRI-WT mice exhibited marked disruption of vascular architecture and diminished fluorescence, whereas UIRI-KO mice restored vascular integrity and enhanced fluorescent signals (Figure 7I, Figure S5J). These results provide direct morphological evidence that *Fbn1* knockout ameliorates capillary rarefaction in UIRI mice.

In order to further clarify the effect of FBN1 on renal vascular endothelial cells, we isolated primary renal endothelial cells from mice. Compared to cells from Sham-WT mice, those from UIRI-WT mice showed robust activation of the ET-1/β-catenin pathway and prominent upregulation of senescence-associated proteins. Conversely, both ET-1/β-catenin pathway activation and senescence-associated protein expression were abrogated in primary endothelial cells from UIRI-KO mice (Figure 7J, Figure S5K). Subsequently, we prepared KTS from different groups and cultured endothelial cells onto these scaffolds to clarify the role of FBN1 in the extracellular microenvironment. Compared with endothelial cells cultured on UIRI-WT KTS, those on UIRI-KO KTS exhibited inhibited activation of the ET-1/β-catenin pathway and reduced expression of senescence-associated proteins (Figure 7K, Figure S5L). Collectively, these results indicate that the FBN1-enriched fibrogenic microenvironment promotes endothelial cell senescence and capillary rarefaction in UIRI mice.

### FBN1 mediates endothelial senescence and microvascular rarefaction in folic acid nephropathy

**Figure 8:**
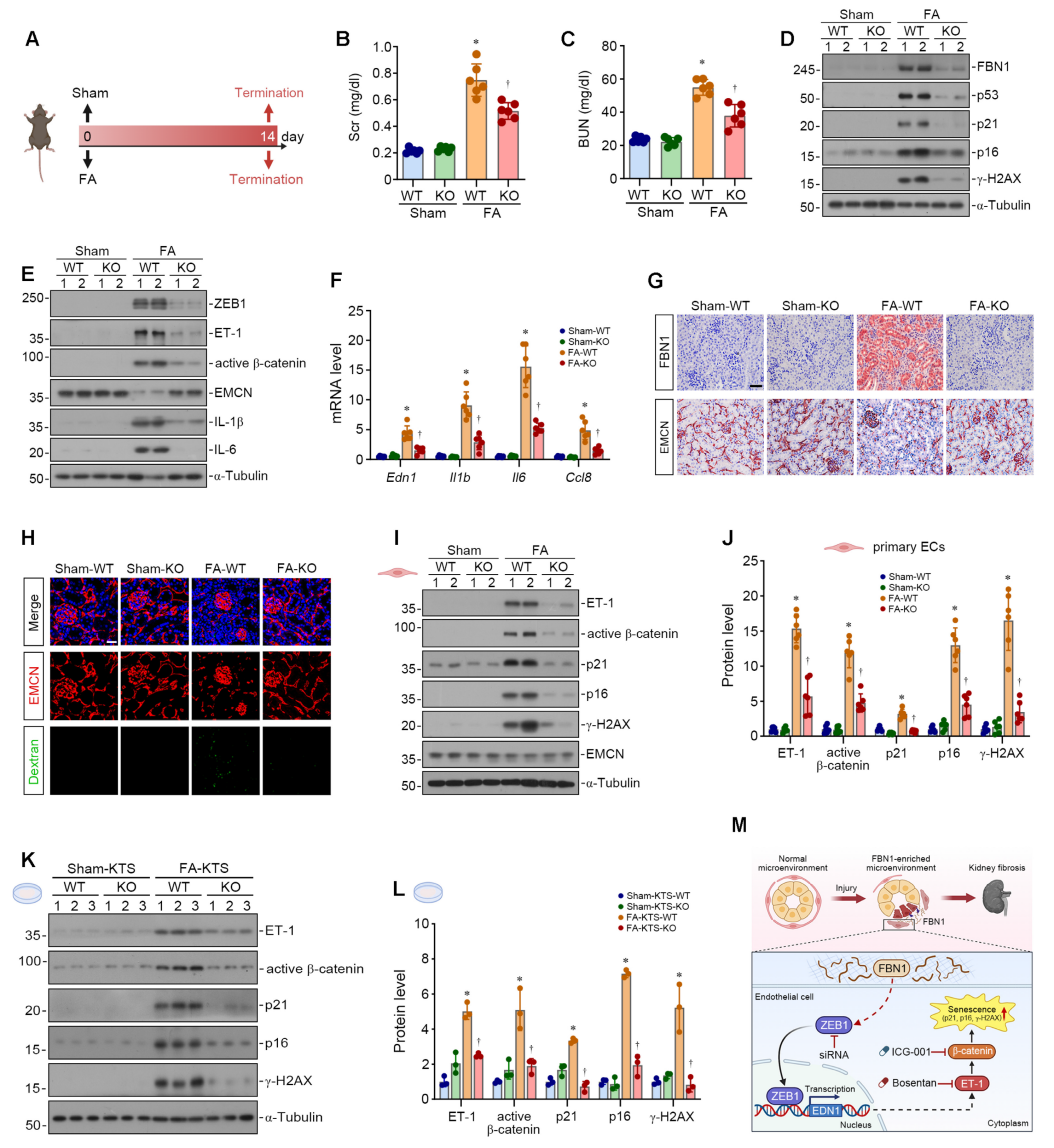
** FBN1 mediates endothelial senescence and microvascular rarefaction in folic acid nephropathy. (A)** The experimental protocol of folic acid (FA) nephropathy model. **(B-C)** Serum creatinine (Scr) and blood urea nitrogen (BUN) levels in different groups as indicated (n = 6). **(D)** Representative Western blot analyses show the renal expression of FBN1, p53, p21, p16 and γ-H2AX proteins in different groups as indicated. **(E)** Representative Western blot analyses show the renal expression of ZEB1, ET-1, active β-catenin, EMCN, IL-1β and IL-6 proteins in different groups as indicated. **(F)** qPCR analyses show the renal mRNA expression levels of *Edn1*, *Il1b*, *Il6*, and *Ccl8*. *P < 0.05 versus Sham-WT, †*P* < 0.05 versus FA-WT (n = 6). **(G)** Representative micrographs show the renal expression and localization of FBN1 and EMCN in different groups by immunohistochemical staining. Scale bar, 50 μm.** (H)** Vascular permeability assay. Representative micrographs show the immunofluorescence staining of EMCN and the fluorescence signals of FITC-dextran. Scale bar, 25 μm.** (I-J)** Representative Western blot (I) and quantitative data (J) show the expression of ET-1, active β-catenin, p21, p16, γ-H2AX and EMCN proteins in different groups of renal primary endothelial cells. *P < 0.05 versus Sham-WT, † *P* < 0.05 versus FA-WT (n = 6). **(K-L)** HUVECs were inoculated on different KTS as indicated. Representative Western blot (K) and quantitative data (L) show the expression of ET-1, active β-catenin, p21, p16 and γ-H2AX proteins in different groups. *P < 0.05 versus Sham-KTS-WT, † *P* < 0.05 versus FA-KTS-WT (n = 3). **(M)** A schematic diagram shows that tubule-derived FBN1 plays a crucial role in orchestrating a pro-senescent microenvironment during renal fibrosis. In this special microenvironment, FBN1 induces endothelial cell senescence via the ZEB1/ET-1/β-catenin pathway, which can be blocked by siZEB1, Bosentan, or ICG-001.

We expanded our studies by using folic acid (FA) nephropathy model. We established a 14-day FA-induced mouse model using WT and KO mice (Figure 8A). The levels of Scr and BUN were increased in FA-WT mice but decreased in FA-KO mice (Figure 8B-C). Western blot analyses demonstrated that *Fbn1* knockout attenuated renal senescence in FA mice (Figure 8D, Figure S6A), inhibited the activation of the ZEB1/ET-1/β-catenin pathway, restored the expression of EMCN protein, and downregulated SASP-related proteins (Figure 8E, Figure S6B). Consistently, *Fbn1* knockout reduced the renal mRNA levels of *Edn1*, *Il1b*, *Il6*, and *Ccl8* in FA mice (Figure 8F). Moreover, IHC staining showed that *Fbn1* knockout markedly reduced FBN1 expression and restored the interstitial capillary density in FA mice (Figure 8G, Figure S6C). FITC-dextran leakage assays revealed diffuse fluorescent signals in the renal interstitium of FA-WT mice, which were markedly attenuated after *Fbn1* knockout, suggesting improved vascular endothelial permeability in FA-induced mice (Figure 8H, Figure S6D-E). Notably, primary renal endothelial cells isolated from FA-KO mice exhibited abolished activation of the ET-1/β-catenin pathway and reduced expression of senescence-associated proteins compared with cells isolated from FA-WT mice (Figure 8I-J). We then inoculated HUVECs onto decellularized KTS prepared from different groups of mice. We found that cells cultured on FA-WT KTS showed robust activation of the ET-1/β-catenin pathway and elevated expression of p21, p16, and γ-H2AX proteins. Conversely, cells cultured on FA-KO KTS displayed inhibited ET-1/β-catenin pathway activation and reduced expression of these senescence-associated proteins (Figure 8K-L). These results indicate that *Fbn1* knockout inhibits endothelial cell senescence and capillary rarefaction in FA mice. In summary, injured renal tubules secrete FBN1 to establish a specific pro-senescent microenvironment in CKD, which promotes endothelial cell senescence and capillary rarefaction by activating the ZEB1/ET-1/β-catenin axis (Figure 8M).

## Discussion

Vascular rarefaction constitutes a key pathological hallmark of renal fibrosis in CKD, yet the molecular mechanisms driving this microvascular attrition remain poorly elucidated 56. This study demonstrates that during CKD progression, renal tubular injury drives pathological secretion of the ECM protein FBN1, which establishes a persistent pro-senescent microenvironment that actively promotes vascular endothelial senescence and renal capillary rarefaction. Using integrated single-cell and spatial transcriptomics, we identified a distinct population of senescent endothelial cells in CKD models showing preferential spatial colocalization with FBN1 deposits. Decellularized scaffold simulations and *in vitro* functional assays confirmed the direct senescence-inducing activity of FBN1. Mechanistically, FBN1 upregulates the transcription factor ZEB1, enabling its binding to the *EDN1* promoter to initiate an ET-1/β-catenin signaling cascade that executes cellular senescence. *ZEB1* knockdown, pharmacological blockade of ET-1 receptors, or β-catenin inhibition each abrogated this pathway. *In vivo*, tubular-specific *Fbn1* knockout significantly improved renal function in multiple murine CKD models while concurrently suppressing endothelial senescence and reducing capillary rarefaction. Vascular ultrasound combined with tissue-clearing-enabled 3D imaging corroborated that *Fbn1* deletion preserved renal microvascular integrity and attenuated rarefaction. Collectively, these findings establish pathological FBN1 deposition as a matrix-encoded signaling hub orchestrating endothelial senescence and capillary attrition during CKD pathogenesis.

This study delineates an active regulatory role for the ECM in governing endothelial senescence. We demonstrate that the ECM, traditionally regarded as merely a passive structural scaffold, functions as a pathogenic orchestrator in disease contexts, with the ECM protein FBN1 serving as a principal paradigm of this pathogenic capacity. By leveraging its intrinsic biochemical properties, including insolubility, high molecular weight, and multi-domain architecture, FBN1 assembles and stabilizes a persistent pro-senescent microenvironment that directly programs endothelial cell senescence. Unlike transient diffusible cytokine signals, this matrix-encoded niche establishes a topologically constrained pathological compartment that entraps endothelial cells and drives irreversible phenotypic transition toward senescence. Therefore, our study provides a novel explanation for the frequent inefficacy of angiogenic factor therapy: pro-senescent microenvironment formation likely constitutes the principal driver of endothelial senescence and vascular rarefaction 57-59. Therapeutic strategies targeting soluble mediators remain unable to reverse the pathologically immobilized matrix deposited around endothelial cells, consequently failing to alter the anchored cellular fate program within this microenvironment. Thus, this work not only identifies a novel signaling axis inducing cellular senescence, but also fundamentally shifts the pathogenic paradigm for CKD-associated capillary rarefaction. By transitioning away from a soluble factor-centric view toward a matrix and niche-aware perspective, this conceptual realignment establishes a new framework for deciphering capillary attrition pathogenesis in CKD.

At the mechanistic level, this study establishes the FBN1/ZEB1/ET-1/β-catenin axis as the principal signaling cascade propagating signals within pro-senescent niches. Specifically, injured tubular epithelial cells aberrantly secrete FBN1, which orchestrates a pathological matrix microenvironment inducing transcriptional upregulation of ZEB1 in endothelial cells. ZEB1 subsequently binds the *EDN1* promoter to enhance transcription, activating the ET-1/β-catenin cascade and executing cellular senescence programs. Notably, while ZEB1 canonically regulates epithelial-mesenchymal transition (EMT) 60, 61, our work demonstrates its functional transcendence in renal fibrosis. Here, ZEB1 serves as a signaling nexus integrating extracellular matrix inputs via FBN1 with intracellular senescence pathways mediated through ET-1/β-catenin. This mechanistic insight expands ZEB1's pathophysiological repertoire in renal disease and aligns with its emerging role as a conserved senescence regulator across tissues. For example, recent systems biology studies identified ZEB1 as a core transcription factor upregulated in aging midbrain neurons, coordinating senescence-associated transcriptional networks 62. Similarly, single-cell analyses in osteoarthritis implicated ZEB1 as a master regulator driving chondrocyte and meniscal senescence, evidenced by *ZEB1* overexpression upregulating genes including *CDKN2A* and *IL6* 63. Collectively, these observations establish ZEB1 as a critical modulator of cellular senescence within specific pathological microenvironments.

Mounting evidence identifies endothelial senescence as both an initiating event and a pivotal driver of systemic aging. Reinforcing this paradigm, a recent human multi-tissue proteomic atlas across the lifespan revealed endothelial cells as a precociously senescent population, which upon entering senescence, secrete a plethora of senescence-associated proteins capable of driving systemic aging 64. Therefore, in the typical pathological scenario of accelerated aging in CKD, the role of endothelial senescence is particularly prominent. Within the evolving CKD microenvironment, endothelial cells likely function as primary sensors of microenvironmental perturbations. Once triggered by pathological stimuli and committed to senescence, these cells not only exhibit cell-autonomous functional impairments including barrier disruption and compromised vascular integrity, but also establish aggressive paracrine signaling through their robust SASP. By continuously releasing inflammatory mediators and matrix-degrading enzymes, this SASP propagates senescence to neighboring cells, amplifies local inflammation, and disrupts vascular homeostasis, thereby consolidating a self-reinforcing vicious cycle 13, 14, 65-67. This pathogenic loop likely underpins the progressive and frequently irreversible trajectory of capillary rarefaction in CKD. Our findings expand this model by demonstrating that FBN1 not merely acts as a critical trigger for this deleterious circuitry, but through its persistent pathological deposition, provides the structural substrate for maintaining the cycle. The sustained accumulation of FBN1 in this niche likely depends on continuous local production. In addition to injured tubular epithelial cells, it is conceivable that fibroblasts and podocytes in the fibrotic kidney represent an accessory source of pathological FBN1 68, further sustaining the pro-senescent niche. This mechanism offers a molecular foundation for understanding the irreversible progression of microvascular decline in CKD.

Beyond inducing senescence, our earlier work documented the capacity of FBN1 to elicit endothelial apoptosis 24. This differential manifestation of senescence versus apoptosis potentially reflects inherent fate-branching processes in endothelial cells responding to FBN1 stimulation, wherein phenotypic trajectory is contingent upon stimulation intensity, exposure duration, or cell-intrinsic status. During sub-lethal chronic FBN1 exposure, the relatively stable senescent phenotype typically predominates. Crucially, whereas apoptosis mediates rapid cellular clearance and immediate resolution, persistent senescent cells actively secrete SASP factors that mediate insidious, cumulative damage within the renal microvasculature. This pathomechanism provides a coherent biological basis explaining the characteristically progressive nature of CKD-associated vascular injury. Nevertheless, irrespective of the endpoint phenotype, FBN1 functions as a paramount upstream regulator perpetuating vascular endothelial injury by establishing a deleterious matrix microenvironment, thereby serving as a primary driver of pathological capillary rarefaction. Of clinical relevance, our prior studies have demonstrated that FBN1 is markedly elevated in kidney biopsies and serum from patients with CKD, where its levels correlate inversely with renal function 24, underscoring the human significance of this pathogenic axis.

Several constraints inherent to this study require explicit acknowledgment. First, the precise identity of cell surface receptor(s) responsible for transducing FBN1-induced senescence signaling in endothelial cells remains elusive. Second, although the FBN1/ZEB1/ET-1/β-catenin axis has been established as a critical pathway governing endothelial senescence, the possibility that FBN1 may cooperatively promote it through additional, parallel mechanisms remains plausible. Third, our data cannot establish the temporal relationship between endothelial senescence and renal fibrosis. Specifically, whether endothelial senescence serves as a causative driver of fibrogenesis or manifests secondary to pre-existing fibrosis remains undetermined. Crucially, these processes likely engage in bidirectional reinforcement, where FBN1-mediated matrix alterations and SASP-dependent signaling pathways mutually potentiate one another, establishing a self-perpetuating pathological circuit. Definitive elucidation will require inducible cell-type-specific lineage tracing coupled with systematic FBN1 interactome mapping.

In conclusion, this work systematically delineates a novel mechanism wherein the ECM protein FBN1 drives endothelial senescence and subsequent capillary rarefaction via activation of the ZEB1/ET-1/β-catenin signaling axis. These results reveal a previously unrecognized pathogenic pathway directly connecting a pro-senescent niche to cellular senescence, while establishing the actively instructive role of FBN1-induced fibrogenic niche in disease progression. Consequently, therapeutic targeting of this pro-senescent microenvironment via FBN1 inhibition, disruption of its downstream signaling hubs, or combination with senotherapeutics represents a promising strategy for preserving renal function, ameliorating vascular attrition, and attenuating CKD progression.

## Supplementary Material

Supplementary figures and tables.

## Data Availability

The data presented in this study will be shared upon reasonable request. The raw transcriptomic data analyzed during this study is available in National Genomics Data Center (BioProject accession number: PRJCA048936).
