# Supplementary material for: Fibrillin-1 Orchestrates a Pro-senescent Niche Driving Peritubular Endothelial Senescence via ZEB1/endothelin-1/β-catenin Signaling
Source: Int J Biol Sci. 2026 May 29;22(11):5911–33. doi: 10.7150/ijbs.133521 (PMC13282740; doi:10.7150/ijbs.133521)
Supplement: Supplementary file 1 — Supplementary figures and tables. [file ijbsv22p5911s1.pdf]

Fibrillin-1 Orchestrates a Pro-senescent Niche Driving Peritubular Endothelial Senescence via ZEB1/endothelin-1/β-catenin Signaling

Junxin Huang[#1,2], Xiaoyao Zhang[#1,2], Zifu Yao[#1,2], Yuxi Zhang[1,2], Di Huang[3], Yongsi Liu[3], Fan Fan Hou[1,2], Youhua Liu[1,2*], Li Li[1,2*]

[1]State Key Laboratory of Multi-organ Injury Prevention and Treatment, National Clinical Research Center for Kidney and Urological Diseases, Division of Nephrology, Nanfang Hospital, Southern Medical University, Guangzhou, China;

[2]Guangdong Provincial Key Laboratory of Renal Failure Research, Guangdong Provincial Institute of Nephrology, Guangzhou, China;

[3]Light Innovation Technology Ltd., Shenzhen, China.

Abstract: 186

Words: 7550

[#]These authors contributed equally.

*To whom correspondence should be addressed:

Li Li, Ph.D or Youhua Liu, Ph.D, Division of Nephrology, Nanfang Hospital, Southern Medical University, 1838 North Guangzhou Avenue, Guangzhou, 510515, China. E-mail: guilinlily3@i.smu.edu.cn or liuyh@smu.edu.cn

Supplementary Figures

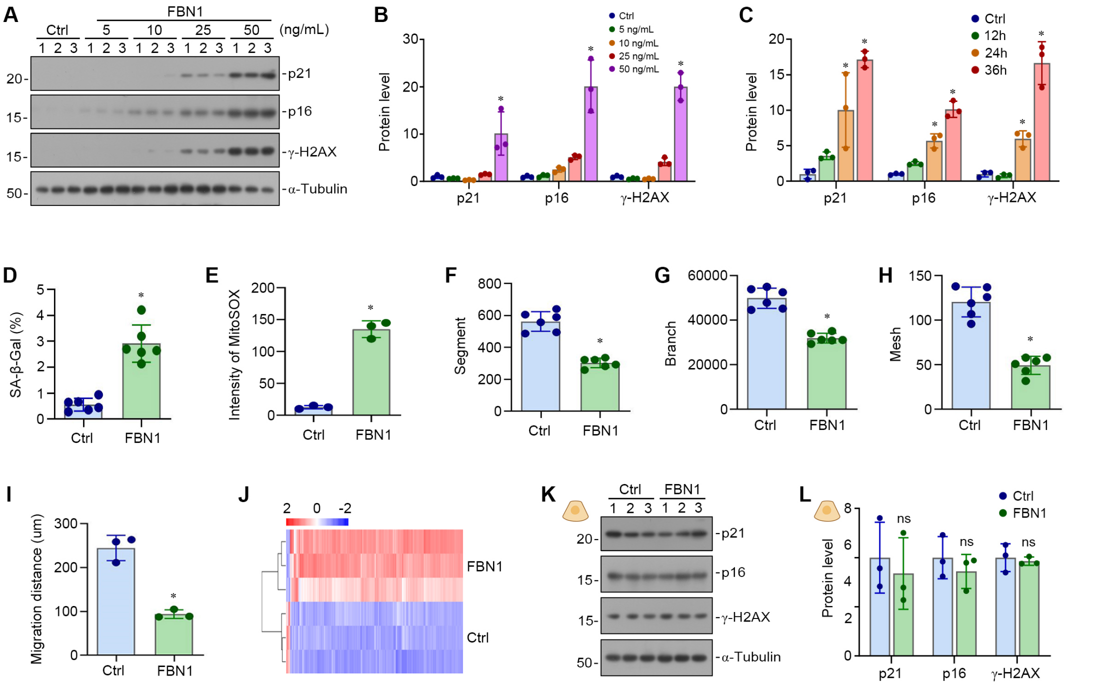

Figure S1. FBN1 induces endothelial cell senescence in vitro. (A) Representative Western blots showing the expression of p21, p16, and γ-H2AX proteins in HUVECs treated with increasing doses of FBN1. **(B)** Quantitative data of p21, p16 and γ-H2AX proteins in HUVECs treated with increasing doses of FBN1. **P* < 0.05 versus Ctrl (n = 3). **(C)** Quantitative data of p21, p16 and γ-H2AX proteins in HUVECs after FBN1 stimulation at different time points. **P* < 0.05 versus Ctrl (n = 3). **(D)** Quantitative data of SA-β-gal positive area in different groups as indicated. **P* < 0.05 versus Ctrl (n = 6). **(E)** Quantitative data of the intensity of MitoSOX staining in different groups as indicated. **P* < 0.05 versus Ctrl (n = 3). **(F-H)** Quantitative data show the levels of segment, branch and mesh in tube formation assays. **P* < 0.05 versus Ctrl (n = 6). **(I)** Quantitative data show the migration distance in different groups. **P* < 0.05 versus Ctrl (n = 3). **(J)** The heatmap shows the gene expression in control cells and FBN1-treated cells. **(K-L)** Representative Western blot (K) and quantitative data (L) show the expression of p21, p16, and γ-H2AX proteins in HK-2 cells treated with FBN1. No significant induction of these proteins was observed (n = 3), indicating that the pro-senescent effect of FBN1 is specific to endothelial cells.

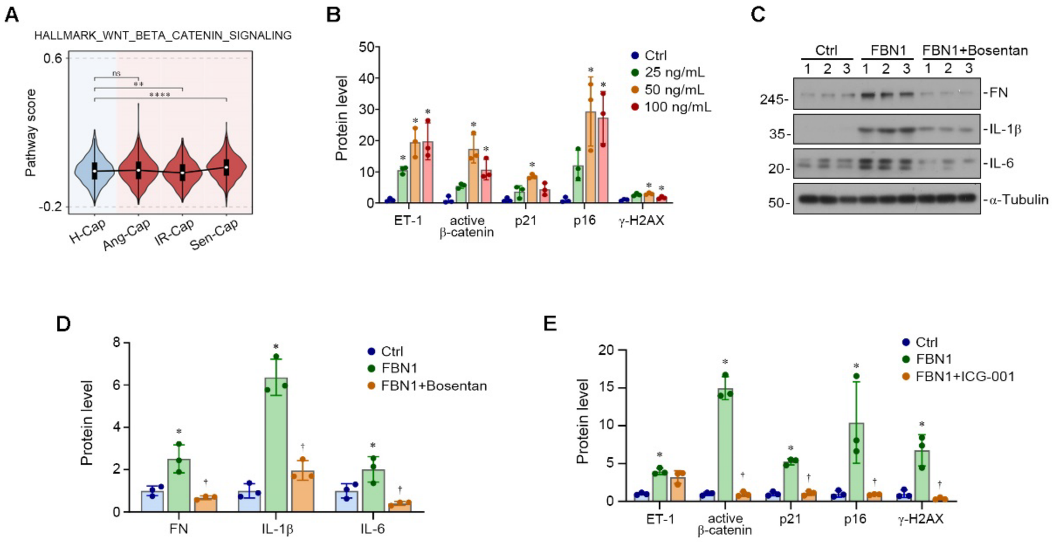

Figure S2. ET-1/β-catenin axis conveys FBN1-derived pro-senescent signaling. (A) Violin plots displaying differences in Wnt/β-catenin signaling scores across endothelial subpopulations. Statistical significance was assessed using the Wilcoxon rank-sum test. ****P < 0.0001. **(B)** Quantitative data of ET-1, active β-catenin, p21, p16 and γ-H2AX proteins in HUVECs treated with different concentrations of ET-1. *P < 0.05 versus Ctrl (n = 3). **(C-D)** Representative Western blot (B) and quantitative data (C) show the expression of FN, IL-1β and IL-6 proteins in different groups as indicated. *P < 0.05 versus Ctrl, †P < 0.05 versus FBN1 (n = 3). **(E)** Quantitative data of ET-1, active β-catenin, p21, p16 and γ-H2AX proteins in different groups as indicated. *P < 0.05 versus Ctrl, †P < 0.05 versus FBN1 (n = 3).

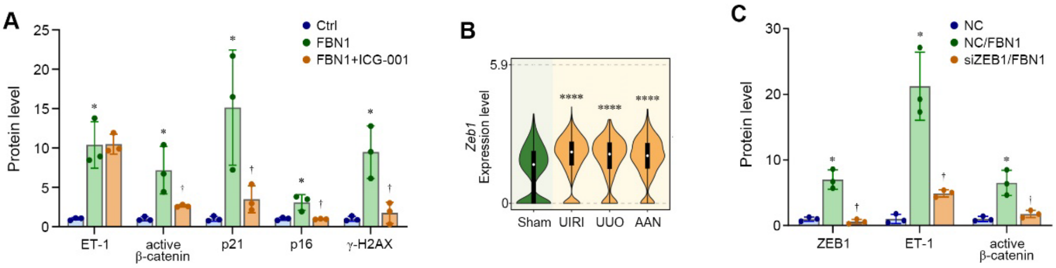

Figure S3. ZEB1 mediates FBN1-induced ET-1/β-catenin activation in endothelial senescence. (A) Quantitative data of ET-1, active β-catenin, p21, p16 and γ-H2AX proteins in primary endothelial cells from different groups. $*P < 0.05$ versus Ctrl, $†P < 0.05$ versus FBN1 (n = 3). **(B)** The expression of *Zeb1* in capillary endothelial cells across three canonical CKD models (UIRI, UUO, AAN). $****P <$ 0.0001 versus Sham. **(C)** Quantitative data of ZEB1, ET-1 and active β-catenin proteins in different groups as indicated. $*P < 0.05$ versus NC, $†P < 0.05$ versus NC/FBN1 (n = 3).

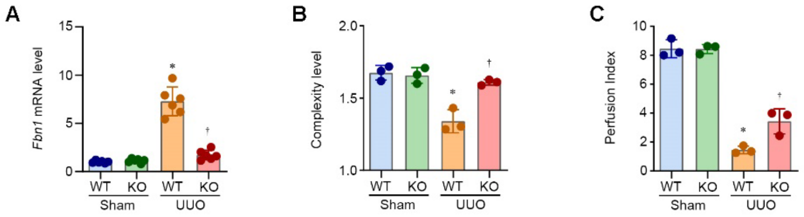

Figure S4. Knockout of *Fbn1* ameliorates microvascular hemodynamics in UUO mice. (A) Quantitative PCR analysis of *Fbn1* mRNA expression in whole kidney. *$P < 0.05$ versus Sham-WT, † $P < 0.05$ versus UUO-WT (n = 6). (**B-C**) Quantitative data of complexity level (B) and perfusion index (C) of microvasculature in different groups as indicated. *$P < 0.05$ versus Sham-WT, †$P < 0.05$ versus UUO-WT (n = 3).

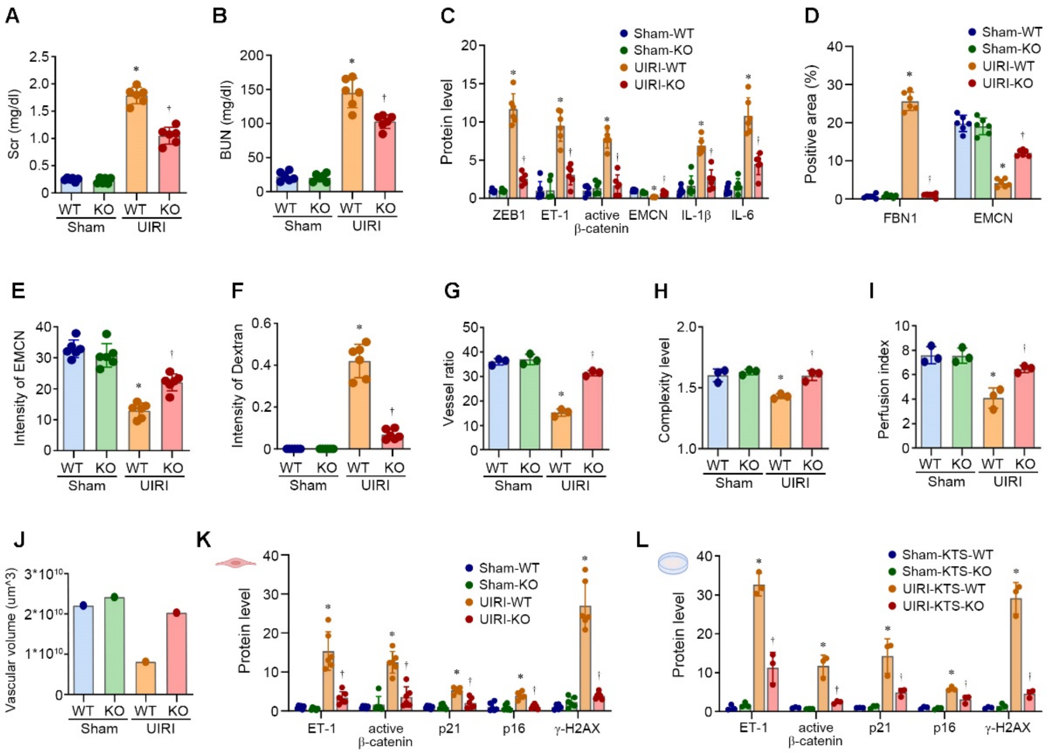

Figure S5. FBN1 drives post-ischemic microvascular decay via senescence acceleration. (A-B) Serum creatinine (Scr) and blood urea nitrogen (BUN) levels in different groups as indicated. *P < 0.05 versus Sham-WT, †P < 0.05 versus UIRI-WT (n = 6). **(C)** Quantitative data of ZEB1, ET-1, active β-catenin, EMCN, IL-1β and IL-6 proteins in different groups as indicated. *P < 0.05 versus Sham-WT, †P < 0.05 versus UIRI-WT (n = 6). **(D)** Quantitative analyses of immunohistochemical staining for FBN1 and EMCN. At least 10 randomly selected microscopic fields were assessed, and the results were averaged for each kidney. *P < 0.05 versus Sham-WT, †P < 0.05 versus UIRI-WT (n = 6). **(E-F)** Quantitative data show the fluorescence intensity of EMCN and FITC-dextran signals. *P < 0.05 versus Sham-WT, †P < 0.05 versus UIRI-WT (n = 6). **(G-I)** Quantitative data show the vessel ratio, complexity level and perfusion index in different groups as indicated. *P < 0.05 versus Sham-WT, †P < 0.05 versus UIRI-WT (n = 3). **(J)** Quantification of vascular spatial volume in cleared kidney tissues (n = 1). **(K)** Quantitative data of ET-1, active β-catenin, p21, p16, γ-H2AX and EMCN proteins in different groups of renal primary endothelial cells. *P < 0.05 versus Sham-WT, †P < 0.05 versus UIRI-WT (n = 6). **(L)** Quantitative data of ET-1, active β-catenin, p21, p16, γ-H2AX and EMCN proteins in HUVECs inoculated on different KTS. *P < 0.05 versus Sham-KTS-WT, †P < 0.05 versus UIRI-KTS-WT (n = 3).

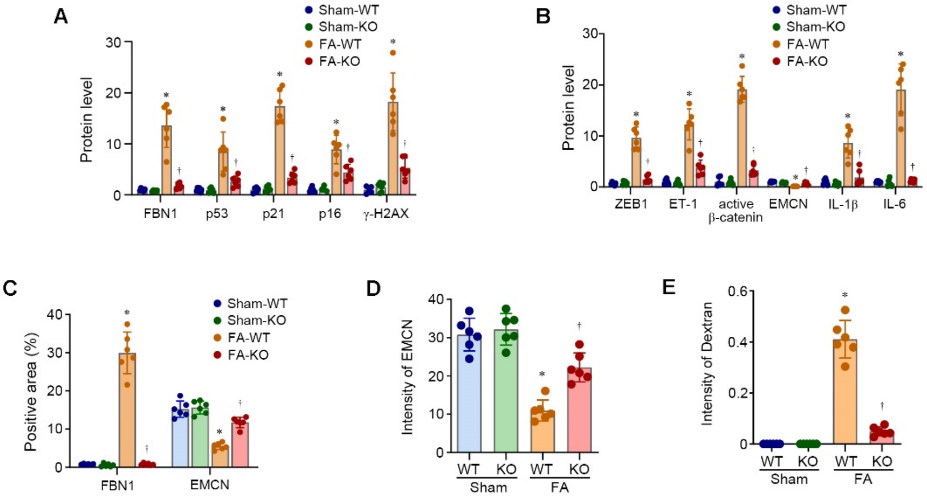

Figure S6. FBN1 mediates endothelial senescence and microvascular rarefaction in folic acid nephropathy. (A) Quantitative data of FBN1, p53, p21, p16 and γ-H2AX proteins in different groups as indicated. **P* < 0.05 versus Sham-WT, †*P* < 0.05 versus FA-WT (n = 6). **(B)** Quantitative data of ZEB1, ET-1, active β-catenin, EMCN, IL-1β and IL-6 proteins in different groups as indicated. **P* < 0.05 versus Sham-WT, †*P* < 0.05 versus FA-WT (n = 6). **(C)** Quantitative analyses of immunohistochemical staining for FBN1 and EMCN. At least 10 randomly selected microscopic fields were assessed, and the results were averaged for each kidney. **P* < 0.05 versus Sham-WT, †*P* < 0.05 versus FA-WT (n = 6). **(D-E)** Quantitative data show the fluorescence intensity of EMCN and FITC-dextran signals. **P* < 0.05 versus Sham-WT, †*P* < 0.05 versus FA-WT (n = 6).

Supplementary Tables

Supplementary Table S1. Publicly available single-cell datasets

GEO Sample	PMID	Mouse Strain	Sex	Age	Modeling methods
GSM5333085	39414946	C57BL/6J	male	12 week	UUO-10D
GSM5333086	39414946	C57BL/6J	male	12 week	UUO-10D
CRX293624	35852860	C57BL/6J	male	7 week	Sham
CRX293625	35852860	C57BL/6J	male	7 week	Sham
CRX293626	35852860	C57BL/6J	male	7 week	Sham
CRX293627	35852860	C57BL/6J	male	7 week	AAN-3W
CRX293628	35852860	C57BL/6J	male	7 week	AAN-3W
CRX293629	35852860	C57BL/6J	male	7 week	AAN-3W
CRA022333	41042124	C57BL/6J	male	8 week	Sham
CRA022333	41042124	C57BL/6J	male	8 week	Sham
CRA022333	41042124	C57BL/6J	male	8 week	UIRI-10D
CRA022333	41042124	C57BL/6J	male	8 week	UIRI-10D

Supplementary Table S2. The sources of antibodies used in this study

**Antibodies**	**Catalogue number**	**Company**	**Location**
**Primary antibodies**			
anti-FBN1	Ab53076	Abcam	Cambridge, MA
anti-FBN1	NBP2-16493	Novus Biologicals	Littleton, CO
anti-γ-H2AX	Ab26350	Abcam	Cambridge, MA
anti-EMCN	AF4666	R & D Systems	Minneapolis, MN
anti-EMCN	A14131	Abclonal	Wuhan, China
anti-p21	A2691	ABclonal	Wuhan, China
anti-p16	Sc-1661	Santa Cruz Biotechnology	Santa Cruz, CA
anti-ET-1	AB2786	Abcam	Cambridge, MA
anti-active β-catenin	19807S	Cell Signaling Technology	Danvers, MA
anti-β-catenin	610154	BD biosciences	San Jose, CA
anti-ZEB1	21544-1-AP	Proteintech Group	Wuhan, China
anti-p53	2524S	Cell Signaling Technology	Danvers, MA
anti-IL-1β	A27676	Abclonal	Wuhan, China
anti-IL-6	A0286	Abclonal	Wuhan, China
anti-FN	F3648	Sigma-Aldrich	St. Louis, MO
anti-α-Tubulin	RM2007	Ray Antibody Biotech	Peachtree Corners, GA
**Secondary antibodies**			
Goat anti-mouse	115-035-003	Jackson Laboratories	West Grove, PA
Goat anti-rabbit	305-035-003	Jackson Laboratories	West Grove, PA
Donkey anti-goat	705-065-147	Jackson Laboratories	West Grove, PA

Supplementary Table S3. Nucleotide sequences of the primers used for qPCR

**Species**	**Gene**	**Primer Sequence 5' to 3'**	
		**Forward**	**Reverse**
Homo	*TNF*	CCTCTCTCTAATCAGCCCTCTG	GAGGACCTGGGAGTAGATGAG
Homo	*IL1B*	ATGATGGCTTATTACAGTGGCAA	GTCGGAGATTCGTAGCTGGA
Homo	*IL6*	ACTCACCTCTTCAGAACGAATTG	CCATCTTTGGAAGGTTCAGGTTG
Homo	*CXCL8*	TTTTGCCAAGGAGTGCTAAAGA	AACCCTCTGCACCCAGTTTTC
Homo	*EDN1*	AAGGCAACAGACCGTGAAAAT	CGACCTGGTTTGTCTTAGGTG
Homo	*ZEB1*	TTACACCTTTGCATACAGAACCC	TTTACGATTACACCCAGACTGC
Homo	*ACTB*	CTCACCATGGATGATGATATCGC	AGGAATCCTTCTGACCCATGC
Mus	*Edn1*	GCACCGGAGCTGAGAATGG	GTGGCAGAAGTAGACACACTC
Mus	*Il1b*	GCAACTGTTCCTGAACTCAACT	ATCTTTTGGGGTCCGTCAACT
Mus	*Il6*	TAGTCCTTCCTACCCCAATTTCC	TTGGTCCTTAGCCACTCCTTC
Mus	*Ccl8*	TCTACGCAGTGCTTCTTTGCC	AAGGGGGATCTTCAGCTTTAGTA
Mus	*Actb*	CAGCTGAGAGGGAAATCGTG	CGTTGCCAATAGTGATGACC
